# Supplementary material for: Genetic Regulation of the 2D to 3D Growth Transition in the Moss Physcomitrella patens
Source: Curr Biol. 2018 Feb 5;28(3):473–478.e5. doi: 10.1016/j.cub.2017.12.052 (PMC5807088; doi:10.1016/j.cub.2017.12.052)
Supplement: Document S1. Figures S1–S4 [file mmc1.pdf]

Current Biology, Volume *28*

Supplemental Information

Genetic Regulation of the 2D to 3D Growth Transition in the Moss *Physcomitrella patens*

Laura A. Moody, Steven Kelly, Ester Rabbinowitsch, and Jane A. Langdale

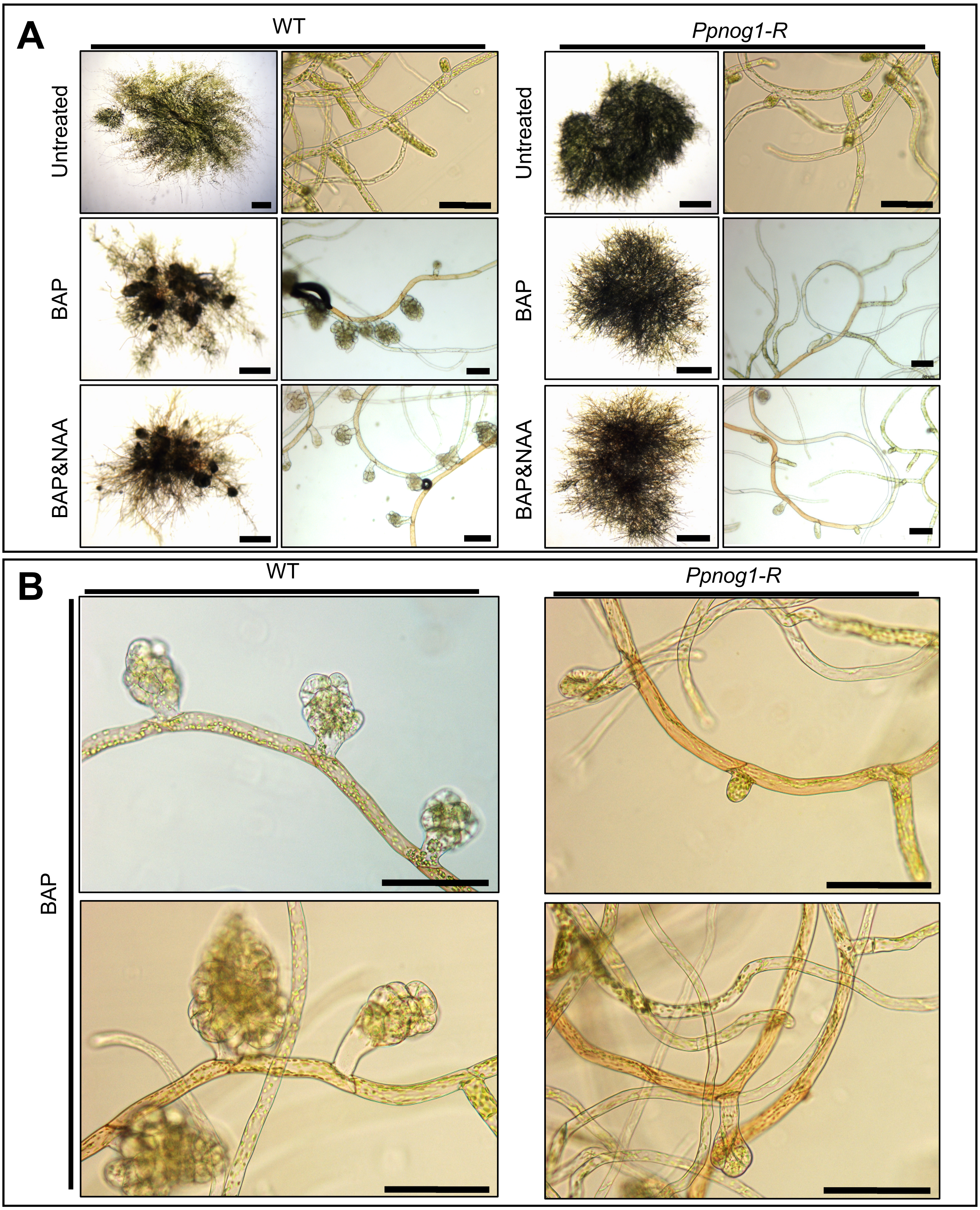

Figure S1. Gametophores are not induced by cytokinin in *Ppnog1-R* mutants. Related to Figure 1. A) 7d old wild-type (WT) and *Ppnog1-R* protonemal filaments cultured in the presence or absence of 1 µm BAP and/or 1 µm NAA. Scale bars = 1 mm. **B)** High magnification images of BAP-treated WT and *Ppnog1-R* protonemal filaments. Scale bars = 50 µm.

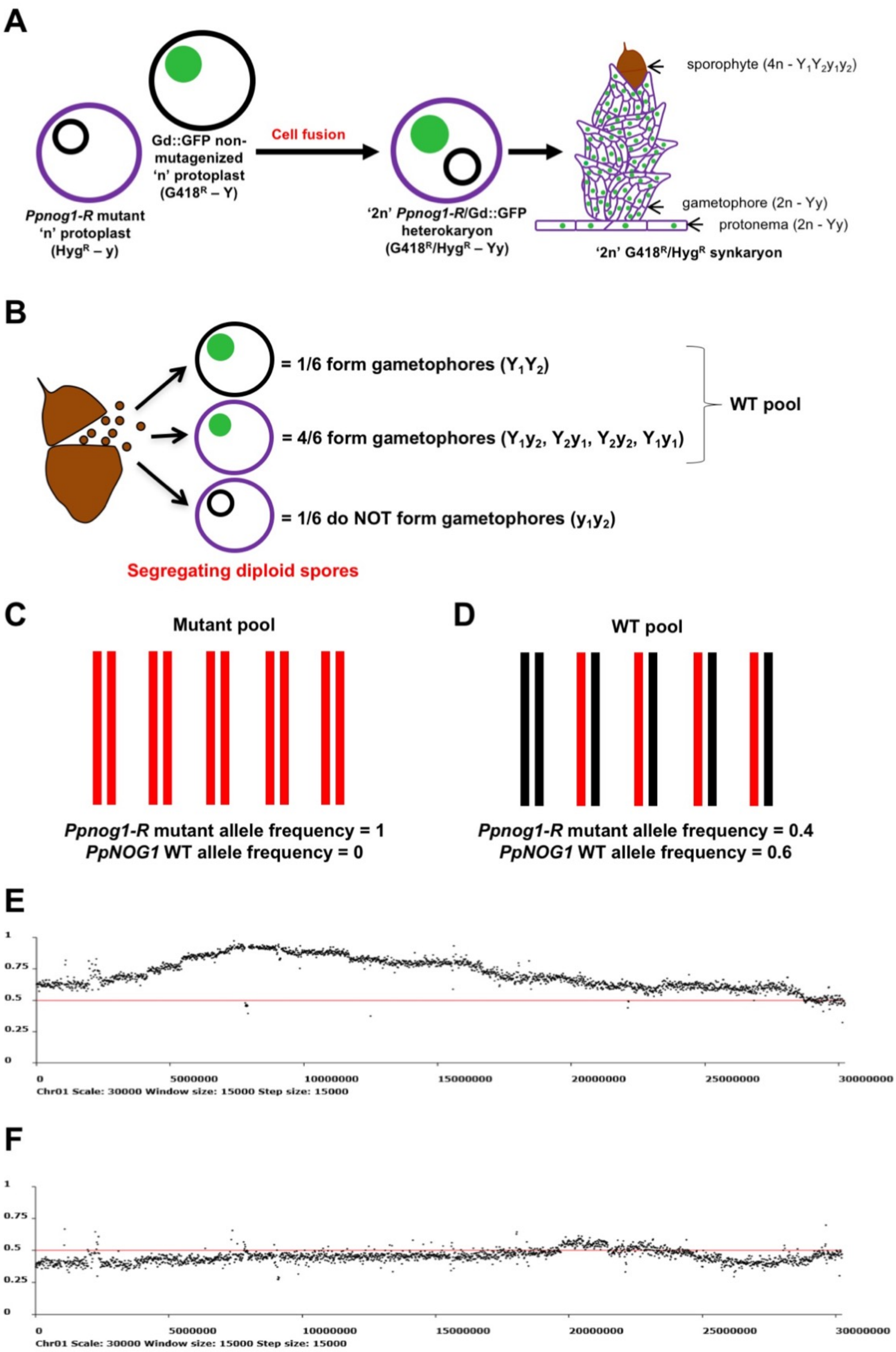

Figure S2. Bulk segregant analysis via somatic hybridization and identification of the *PpNOG1* locus. Related to Figure 2. A) Somatic hybridization between reproductively sterile *Ppnog1-R* (haploid, n) and Gd::GFP (n) protoplasts yields viable *Ppnog1-R*/Gd::GFP diploid hybrid protoplasts which regenerate to form diploid protonema and gametophores, that produce tetraploid sporophytes after fertilization. **B)** *Ppnog1-R*/Gd::GFP tetraploid sporophyte undergoes meiosis to produce phenotypically segregating diploid spores. **C, D)** Segregating progeny sequenced in separate mutant (C) and wild-type pools (D). Expected *Ppnog1-R* and *NOG1* allele frequencies in each pool are indicated. **E, F)** Allele frequency plots for *Ppnog1-R* (E) and wild-type (F) segregants along chromosome 1 of *Physcomitrella patens*.

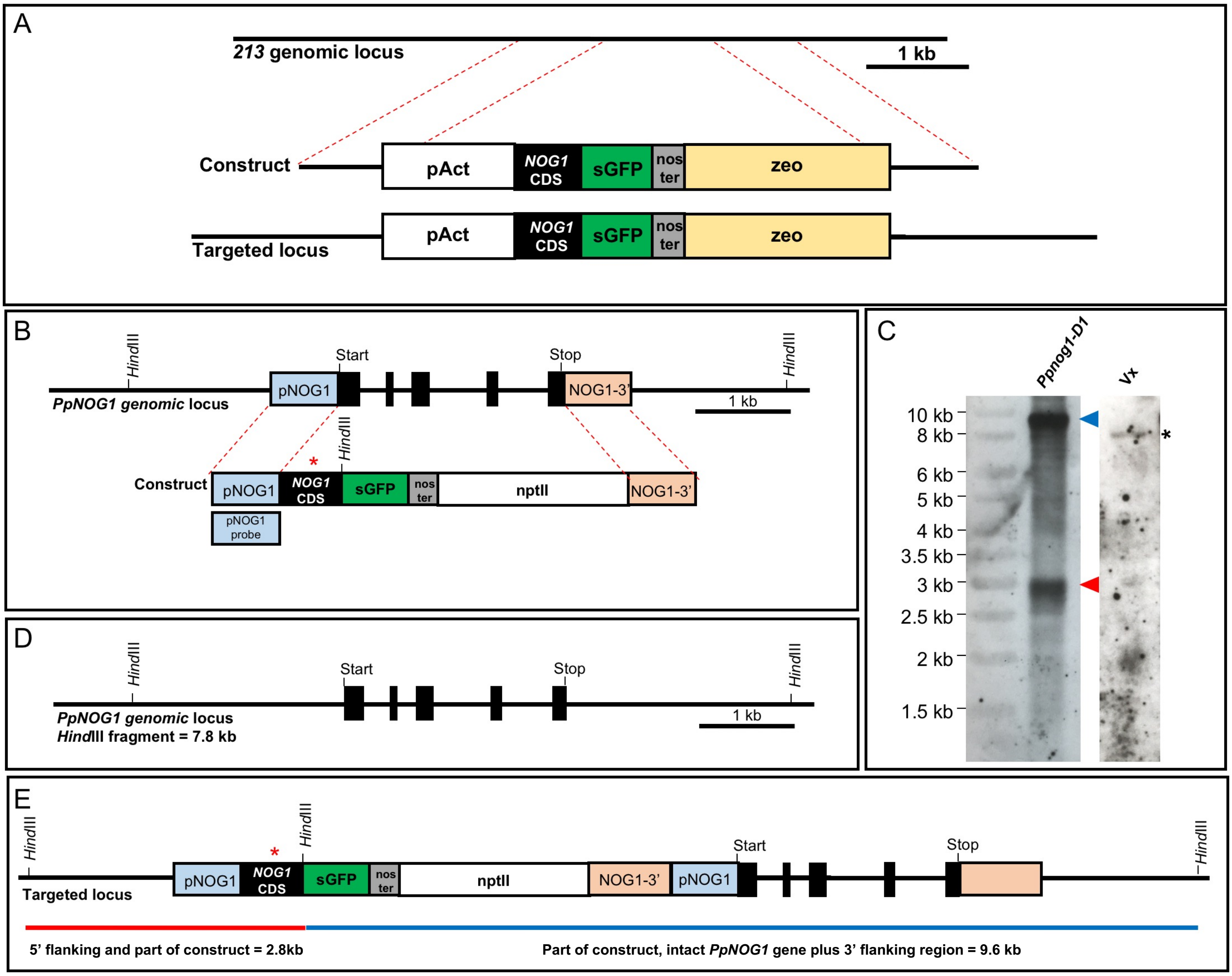

Figure S3. Generation of *PpNOG1* complementation and disruptant lines. Related to Figure 3. A) Schematic of the construct used to complement the *Ppnog1-R* mutant phenotype. **B)** Schematic of the construct used to disrupt the *PpNOG1* locus in the Vx parental line. The *pNOG1* probe used in (C) is indicated. **C)** DNA gel blot analysis of the disruptant line. Genomic DNA from Vx parental and *Ppnog1-D1* disruptant lines was digested with HindIII and hybridized with the *pNOG1* probe (B). An asterisk (*) denotes the presence of an intact *PpNOG1* locus in Vx (detailed in D) and coloured arrows refer to two separate fragments from the disrupted *PpNOG1* locus (detailed in E). **D)** Schematic of the *PpNOG1* locus in parental Vx (* in C). **E)** Schematic of *PpNOG1* locus in the disruptant line *Ppnog1-D1* (coloured arrows in C). Expected fragment sizes are indicated, which are supported by sequencing data (data not shown).

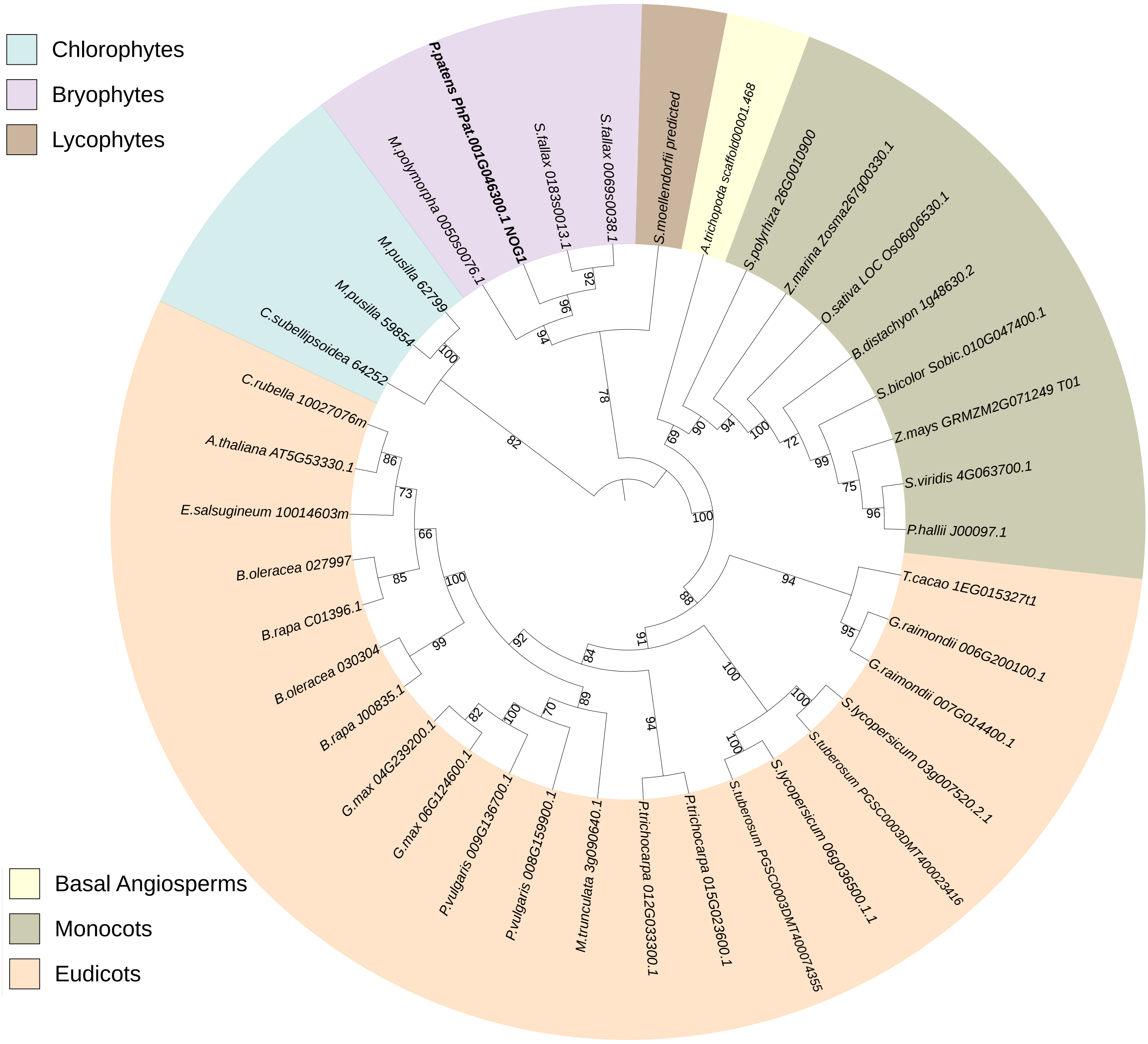

Figure S4. Maximum-likelihood phylogenetic analysis of NOG1 homologs. Related to Figure 4. Bootstrap values are indicated next to the corresponding branch. Colour coded boxes correspond to the species phyla. The tree was rooted using sequences from the chlorophyte clade that share homology in the ubiquitin-associated domain but are otherwise dissimilar (see Data S4 for sequence alignment).
