# Supplementary material for: Genetic Regulation of the 2D to 3D Growth Transition in the Moss Physcomitrella patens
Source: Curr Biol. 2018 Feb 5;28(3):473–478.e5. doi: 10.1016/j.cub.2017.12.052 (PMC5807088; doi:10.1016/j.cub.2017.12.052)
Supplement: Data S1. Sequences of PpNOG1 Complementation (A) and Disruptant (B) Constructs, Related to Figure 3 [file mmc2.docx]

**DATA S1.**

**A) *PpNOG1* complementation construct.**

PpNOG1_CDS in red

mGFP in green

>pAct:*PpNOG1*-mGFP

TCCCTTTTTTGCGGCATTTTGCCTTCCTGTTTTTGCTCACCCAGAAACGCTGGTGAAAGTAAAAGATGCTGAAGATCAGTTGGGTGCACGAGTGGGTTACATCGAACTGGATCTCAACAGCGGTAAGATCCTTGAGAGTTTTCGCCCCGAAGAACGTTTTCCAATGATGAGCACTTTTAAAGTTCTGCTATGTGGCGCGGTATTATCCCGTATTGACGCCGGGCAAGAGCAACTCGGTCGCCGCATACACTATTCTCAGAATGACTTGGTTGAGTACTCACCAGTCACAGAAAAGCATCTTACGGATGGCATGACAGTAAGAGAATTATGCAGTGCTGCCATAACCATGAGTGATAACACTGCGGCCAACTTACTTCTGACAACGATCGGAGGACCGAAGGAGCTAACCGCTTTTTTGCACAACATGGGGGATCATGTAACTCGCCTTGATCGTTGGGAACCGGAGCTGAATGAAGCCATACCAAACGACGAGCGTGACACCACGATGCCTGTAGCAATGGCAACAACGTTGCGCAAACTATTAACTGGCGAACTACTTACTCTAGCTTCCCGGCAACAATTAATAGACTGGATGGAGGCGGATAAAGTTGCAGGACCACTTCTGCGCTCGGCCCTTCCGGCTGGCTGGTTTATTGCTGATAAATCTGGAGCCGGTGAGCGTGGGTCTCGCGGTATCATTGCAGCACTGGGGCCAGATGGTAAGCCCTCCCGTATCGTAGTTATCTACACGACGGGGAGTCAGGCAACTATGGATGAACGAAATAGACAGATCGCTGAGATAGGTGCCTCACTGATTAAGCATTGGTAACTGTCAGACCAAGTTTACTCATATATACTTTAGATTGATTTAAAACTTCATTTTTAATTTAAAAGGATCTAGGTGAAGATCCTTTTTGATAATCTCATGACCAAAATCCCTTAACGTGAGTTTTCGTTCCACTGAGCGTCAGACCCCGTAGAAAAGATCAAAGGATCTTCTTGAGATCCTTTTTTTCTGCGCGTAATCTGCTGCTTGCAAACAAAAAAACCACCGCTACCAGCGGTGGTTTGTTTGCCGGATCAAGAGCTACCAACTCTTTTTCCGAAGGTAACTGGCTTCAGCAGAGCGCAGATACCAAATACTGTCCTTCTAGTGTAGCCGTAGTTAGGCCACCACTTCAAGAACTCTGTAGCACCGCCTACATACCTCGCTCTGCTAATCCTGTTACCAGTGGCTGCTGCCAGTGGCGATAAGTCGTGTCTTACCGGGTTGGACTCAAGACGATAGTTACCGGATAAGGCGCAGCGGTCGGGCTGAACGGGGGGTTCGTGCACACAGCCCAGCTTGGAGCGAACGACCTACACCGAACTGAGATACCTACAGCGTGAGCTATGAGAAAGCGCCACGCTTCCCGAAGGGAGAAAGGCGGACAGGTATCCGGTAAGCGGCAGGGTCGGAACAGGAGAGCGCACGAGGGAGCTTCCAGGGGGAAACGCCTGGTATCTTTATAGTCCTGTCGGGTTTCGCCACCTCTGACTTGAGCGTCGATTTTTGTGATGCTCGTCAGGGGGGCGGAGCCTATGGAAAAACGCCAGCAACGCGGCCTTTTTACGGTTCCTGGCCTTTTGCTGGCCTTTTGCTCACATGTTCTTTCCTGCGTTATCCCCTGATTCTGTGGATAACCGTATTACCGCCTTTGAGTGAGCTGATACCGCTCGCCGCAGCCGAACGACCGAGCGCAGCGAGTCAGTGAGCGAGGAAGCGGAAGAGCGCCCAATACGCAAACCGCCTCTCCCCGCGCGTTGGCCGATTCATTAATGCAGCTGGCACGACAGGTTTCCCGACTGGAAAAGCGGGCAGTGAGCGCAACGAATTAATGTGAGTTAGCTCACTCATTAGGCACCCCAGGCTTTACACTTTATGCTCCCGGCTCGTATGTTGTGTGGAATTGTGAGCGGATAACAATTTCACACAGGAAACAGCTATGACCATGATTACGCCAAGCGCGCAATTAACCCTCACTAAAGGGAACAAAAGCTGGGTACCATCAGACGTAAATATAAAGTTGTAAGGAAAATTAGTTCTATAAAATGCCTCTATATTAACATTCAATTGTAAAGGTGCTGCGTATTCATGGATTAGTATATAAATTATTATTTTTGTAAAATGTGTGTGTGTGTTGTTAATGCTTTATGTAAAAAGAACTGTGTGCTTCAAAATACTATAGATCCCTTTTAGACAGATTATCTTTATGTAAAAAGAACTGTGTGCTTCAAAATACTATAGATCCCTTTTAGACAGAATATCTATAATATGTAGAAGTTAAATTCGGTTTTAATTCTGTACCATGTATTGTGAACATCTAGATTTTGTGATACAGCTTCATGAAAAACTTCAAAACTTCAAAACATTTTACTTCTATAAAGTTATCAATTTGTTTGACAAACAACCTTATATGGTAATGACAAGAGTCTCACATTAATTCAGCATCTCAAAAATAAATATAAATACTCTTTTATACTCAAAATTGTATTTAATACAAAACATATGCATGTATCAATCCAATGACTTGGGCCAATAATGTAAGTCACTCATTATAGTATTAGTATTTATTATTCCTTTTTGTTTCTCAGCAAAAAAAAAAAAAAAAAAAAAAAAAAAAAAAAACTCATCTATGTGATTCGTTCTCAACACTTACACTTCAAAAATATTTATTTACTCATTCATCAATACATCCAAGATAAAATATATATATATAGATATATATATATATATATATATATATTTATCTTTTTCTAACCTCTTCTACAGAGTTTTGTTCAGTGTTGTAAACCCAAACTTGGTGTCGAGGGCCCGAGGTCATTCATATGCTTGAGAAGAGAGTCGGGATAGTCCAAAATAAAACAAAGGTAAGATTACCTGGTCAAAAGTGAAAACATCAGTTAAAAGGTGGTATAAAGTAAAATATCGGTAATAAAAGGTGGCCCAAAGTGAAATTTACTCTTTTCTACTATTATAAAAATTGAGGATGTTTTTGTCGGTACTTTGATACGTCATTTTTGTATGAATTGGTTTTTAAGTTTATTCGCTTTTGGAAATGCATATCTGTATTTGAGTCGGGTTTTAAGTTCGTTTGCTTTTGTAAATACAGAGGGATTTGTATAAGAAATATCTTTAAAAAAACCCATATGCTAATTTGACATAATTTTTGAGAAAAATATATATTCAGGCGAATTCTCACAATGAACAATAATAAGATTAAAATAAGCTTTCCCCCGTTGCAGCGCATGGGTATTTTTTCTAGTAAAAATAAAAGATAAACTTAGACTCAAAACATTTACAAAAACAACCCCTAAAGTTCCTAAAGCCCAAAGTGCTATCCACGATCCATAGCAAGCCCAGCCCAACCCAACCCAACCCAACCCACCCCAGTCCAGCCAACTGGACAATAGTCTCCACACCCCCCCACTATCACCGTGAGTTGTCCGCACGCACCGCACGTCTCGCAGCCAAAAAAAAAAAAGAAAGAAAAAAAAGAAAAAGAAAAAACAGCAGGTGGGTCCGGGTCGTGGGGGCCGGAAACGCGAGGAGGATCGCGAGCCAGCGACGAGGCCGGCCCTCCCTCCGCTTCCAAAGAAACGCCCCCCATCGCCACTATATACATACCCCCCCCTCTCCTCCCATCCCCCCAACCCTACCACCACCACCACCACCACCTCCACCTCCTCCCCCCTCGCTGCCGGACGACGAGCTCCTCCCCCCTCCCCCTCCGCCGCCGCCGCGCCGGTAACCACCCCGCCCCTCTCCTCTTTCTTTCTCCGTTTTTTTTTTCCGTCTCGGTCTCGATCTTTGGCCTTGGTAGTTTGGGTGGGCGAGAGGCGGCTTCGTGCGCGCCCAGATCGGTGCGCGGGAGGGGCGGGATCTCGCGGCTGGGGCTCTCGCCGGCGTGGATCCGGCCCGGATCTCGCGGGGAATGGGGCTCTCGGATGTAGATCTGCGATCCGCCGTTGTTGGGGGAGATGATGGGGGGTTTAAAATTTCCGCCATGCTAAACAAGATCAGGAAGAGGGGAAAAGGGCACTATGGTTTATATTTTTATATATTTCTGCTGCTTCGTCAGGCTTAGATGTGCTAGATCTTTCTTTCTTCTTTTTGTGGGTAGAATTTGAATCCCTCAGCATTGTTCATCGGTAGTTTTTCTTTTCATGATTTGTGACAAATGCAGCCTCGTGCGGAGCTTTTTTGTAGGTAGAAGATATCGGGGATCCTCTAGAGTCGACATGGAATATGATTATGGACGAAGCGGGCACGGGAGTGGTGGTTATGAGATGGGAAGGCCGATGTATCATAGTAGACAAGGGAGTAACGTGCAAGGTAGCTATCCCCGAGTAGGGCAATCCGCAGGGGATGCATTGATGAATCGCGGTCCGCCCCAGGCACCTCTTTTATCGGTTCCATCTTTCCCTTCAGGATCAGCTATCAAAGTGACCATCAAACCTATGTATCGATTGGGTCCACCTGCTCAACTAAGAGTGCAGAGCAGGGAGGTGCCCCGGAGCTTATTTCAGTTCGAGTTTGATTTAGAGCGTCGCATCCTTGCAGAGGCGGAGCAGGGAAATCTCAATTTTAGAGCTGGAAGTGGGGCCACTCTAAGTCAATCTAACTCGGAAGCTGATCTTGCTGAAGTGGAAGATGCGACGGTAGCCAAATATCTTGCCATGGGCCATAATAAAGAAGCTGTACAGTATGCTCTTCAAACTTATGGAGACGATCAAAACAAGGTTCTTGATTTCTGTCCTCCTTTCAATAGAATTCGAGAAATGGGGTTTGCAGCCGATCGTGTTGCAAAGGCCTTGGCCAGCTGTAATAATGATGAGGAGCAAGCAATATCATCTCTAGTTTCTAAGCTTTTGGTGAGCAAGGGCGAGGAGCTGTTCACCGGGGTGGTGCCCATCCTGGTCGAGCTGGACGGCGACGTAAACGGCCACAAGTTCAGCGTGTCCGGCGAGGGCGAGGGCGATGCCACCTACGGCAAGCTGACCCTGAAGTTCATCTGCACCACCGGCAAGCTGCCCGTGCCCTGGCCCACCCTCGTGACCACCTTCACCTACGGCGTGCAGTGCTTCAGCCGCTACCCCGACCACATGAAGCAGCACGACTTCTTCAAGTCCGCCATGCCCGAAGGCTACGTCCAGGAGCGCACCATCTTCTTCAAGGACGACGGCAACTACAAGACCCGCGCCGAGGTGAAGTTCGAGGGCGACACCCTGGTGAACCGCATCGAGCTGAAGGGCATCGACTTCAAGGAGGACGGCAACATCCTGGGGCACAAGCTGGAGTACAACTACAACAGCCACAACGTCTATATCATGGCCGACAAGCAGAAGAACGGCATCAAGGTGAACTTCAAGATCCGCCACAACATCGAGGACGGCAGCGTGCAGCTCGCCGACCACTACCAGCAGAACACCCCCATCGGCGACGGCCCCGTGCTGCTGCCCGACAACCACTACCTGAGCACCCAGTCCGCCCTGAGCAAAGACCCCAACGAGAAGCGCGATCACATGGTCCTGCTGGAGTTCGTGACCGCCGCCGGGATCACTCACGGCATGGACGAGCTGTACAAGTAAAGCGGCCGATCGTTCAAACATTTGGCAATAAAGTTTCTTAAGATTGAATCCTGTTGCCGGTCTTGCGATGATTATCATATAATTTCTGTTGAATTACGTTAAGCATGTAATAATTAACATGTAATGCATGACGTTATTTATGAGATGGGTTTTTATGATTAGAGTCCCGCAATTATACATTTAATACGCGATAGAAAACAAAATATAGCGCGCAAACTAGGATAAATTATCGCGCGCGGTGTCATCTATGTTACTAGATCCGATGATAAGCTGTCAAACATGAGAATTCCCTTTCAGAAAGAATGCTAACCCACAGATGGTTAGAGAGGCTTACGCAGCAGGTCTCATCAAGACGATCTACCCGAGCAATAATCTCCAGGAAATCAAATACCTTCCCAAGAAGGTTAAAGATGCAGTCAAAAGATTCAGGACTAACTGCATCAAGAACACAGAGAAAGATATATTTCTCAAGATCAGAAGTACTATTCCAGTATGGACGATTCAAGGCTTGCTTCACAAACCAAGGCAAGTAATAGAGATTGGAGTCTCTAAAAAGGTAGTTCCCACTGAATCAAAGGCCATGGAGTCAAAGATTCAAATAGAGGACCTAACAGAACTCGCCGTAAAGACTGGCGAACAGTTCATACAGAGTCTCTTACGACTCAATGACAAGAAGAAAATCTTCGTCAACATGGTGGAGCACGACACACTTGTCTACTCCAAAAATATCAAAGATACAGTCTCAGAAGACCAAAGGGCAATTGAGACTTTTCAACAAAGGGTAATATCCGGAAACCTCCTCGGATTCCATTGCCCAGCTATCTGTCACTTTATTGTGAAGATAGTGGAAAAGGAAGGTGGCTCCTACAAATGCCATCATTGCGATAAAGGAAAGGCCATCGTTGAAGATGCCTCTGCCGACAGTGGTCCCAAAGATGGACCCCCACCCACGAGGAGCATCGTGGAAAAAGAAGACGTTCCAACCACGTCTTCAAAGCAAGTGGATTGATGTGATATCTCCACTGACGTAAGGGATGACGCACAATCCCACTATCCTTCGCAAGACCCTTCCTCTATATAAGGAAGTTCATTTCATTTGGAGAGGGGATCCATGGCCAAGTTGACCAGTGCCGTTCCGGTGCTCACCGCGCGCGACGTCGCCGGAGCGGTCGAGTTCTGGACCGACCGGCTCGGGTTCTCCCGGGACTTCGTGGAGGACGACTTCGCCGGTGTGGTCCGGGACGACGTGACCCTGTTCATCAGCGCGGTCCAGGACCAGGTGGTGCCGGACAACACCCTGGCCTGGGTGTTGGTGCGCGGCCTGGACGAGCTGTACGCCGAGTGGTCGGAGGTCGTGTCCACGAACTTCCGGGACGCCTCCGGGCCGGCCATGACCGAGATCGGCGAGCAGCCGTGGGGGCGGGAGTTCGCCCTGCGCGACCCGGCCGGCAACTGCGTGCACTTCGTGGCCGAGGAGCAGGACTGAGCGGGACTCTGGGGTTCGGACGGTACGCTGAAATCACCAGTCTCTCTCTACAAATCTATCTCTCTCTATTTTCTCCATAAATAATGTGTGAGTAGTTTCCCGATAAGGGAAATTAGGGTTCTTATAGGGTTTCGCTCATGTGTTGAGCATATAAGAAACCCTTAGTATGTATTTGTATTTGTAAAATACTTCTATCAATAAAATTTCTAATTCCTAAAACCAAAATCCAGTACTAAAATCCAGATCTCCTAAAGTCCCTATAGATCTTTGTCGTGAATATAAACCAGACACGAGACGACTAAACCTGGAGCCCAGACGCCGTTCGAAGCTAGAAGTACCGCTTAGGCAGGAGGCCGTTAGGGAAAAGATGCTAAGGCAGGGTTGGTTACGTTGACTCCCCCGTAGGTTTGGTTTAAATATGATGAAGTGGACGGAAGGAAGGAGGAAGACAAGGAAGGATAAGGTTGCAGGCCCTGTGCAAGGTAAGAAGATGGAAATTTGATAGAGGTACGCTACTATACTTATACTATACGCTAAGGGAATGCTTGTATTTATACCCTATACCCCCTAATAACCCCTTATCAATTTAAGAAATAATCCGCATAAGCCCCCGCTTAAAAATTGGTATCAGAGCCATGAATAGGTCTATGACCAAAACTCAAGAGGATAAAACCTCACCAAAATACGAAAGAGTTCTTAACTCTAAAGATAAAAGATCTTTCAAGATCAAAACTAGTTCCCTCACACCGGTGACGGGGATCCACTAGTTCTAGAGCGGCCGCAACTTAGAAGTAGCGTGTTTCTCTACATCACGCTTTTATCCCTTTGTAAATTTTAGAGCGTAGGTTGGCATGGGGGGGCTATTGGCTGTGAAAGGGACAGAGATATCTGCTGCACCATGTTCTGTACATAAAGCTGCCGCTCTGTTACGCCGTTTCTTGTGAACCGAGGCAGCTTCTGAGGTTGGCTTTGCTTAAAGTTGCGCTGATGCTGTTTCCCAATGTTTAGAGGAAATCCTGGTGACGAGAACGAAGAACTCGGGAATTACTAGGGAGGATTTGAGGTTTGTATCTGCAGGGCGTGGTTCAAAATTCTTTCATGTCGAACAGGTTGACAAAGTTCCAGAAATTATGAAATCTCAAAGAGGTAAAGGGTCGAAACGTTTGAAGATCTCGAAAGAAAAAGGAGGAGAAAGGTTGAACATCATTTCATTTTAAGGCATTTCGAGGACATAAAATTGGATGGTGCAGAGGGGAGAAAGCAGGCCAATGGGAAGCTAGGTGTCGATGAAAAGCCTGTCGTTACCGAATTGCTTTCGAGTACACCTAGGCAATGAGATCCAGAAGATAATTACAGAGAATTTCAAAAGAATGAAAAGATGAAGAAAAAGAAGAAGAGTTTGAATGGAGAGGCAATGGATGTGGATGTAGGAGGGTTGTACTCGGACATTTTGATCGAGGCCGATGTGGATTGGTACCCTGATATTACGAAAAACATTGTAGAGCATCTGACATAATGTCTTCTAAAGTACCGCGGTGGAGCTCCAATTCGCCCTATAGTGAGTCGTATTACGCGCGCTCACTGGCCGTCGTTTTACAACGTCGTGACTGGGAAAACCCTGGCGTTACCCAACTTAATCGCCTTGCAGCACATCCCCCTTTCGCCAGCTGGCGTAATAGCGAAGAGGCCCGCACCGATCGCCCTTCCCAACAGTTGCGCAGCCTGAATGGCGAATGGGACGCGCCCTGTAGCGGCGCATTAAGCGCGGCGGGTGTGGTGGTTACGCGCAGCGTGACCGCTACACTTGCCAGCGCCCTAGCGCCCGCTCCTTTCGCTTTCTTCCCTTCCTTTCTCGCCACGTTCGCCGGCTTTCCCCGTCAAGCTCTAAATCGGGGGCTCCCTTTAGGGTTCCGATTTAGTGCTTTACGGCACCTCGACCCCAAAAAACTTGATTAGGGTGATGGTTCACGTAGTGGGCCATCGCCCTGATAGACGGTTTTTCGCCCTTTGACGTTGGAGTCCACGTTCTTTAATAGTGGACTCTTGTTCCAAACTGGAACAACACTCAACCCTATCTCGGTCTATTCTTTTGATTTATAAGGGATTTTGCCGATTTCGGCCTATTGGTTAAAAAATGAGCTGATTTAACAAAAATTTAACGCGAATTTTAACAAAATATTAACGCTTACAATTTAG

**B) *PpNOG1* disruptant construct**

PpNOG1_CDS in red

sGFP in green

>*pNOG1*:*PpNOG1**-mGFPmutNPTII-3’

CtaaattgtaagcgttaatattttgttaaaattcgcgttaaatttttgttaaatcagctcattttttaaccaataggccgaaatcggcaaaatcccttataaatcaaaagaatagaccgagatagggttgagtgttgttccagtttggaacaagagtccactattaaagaacgtggactccaacgtcaaagggcgaaaaaccgtctatcagggcgatggcccactacgtgaaccatcaccctaatcaagttttttggggtcgaggtgccgtaaagcactaaatcggaaccctaaagggagcccccgatttagagcttgacggggaaagccggcgaacgtggcgagaaaggaagggaagaaagcgaaaggagcgggcgctagggcgctggcaagtgtagcggtcacgctgcgcgtaaccaccacacccgccgcgcttaatgcgccgctacagggcgcgtcccattcgccattcaggctgcgcaactgttgggaagggcgatcggtgcgggcctcttcgctattacgccagctggcgaaagggggatgtgctgcaaggcgattaagttgggtaacgccagggttttcccagtcacgacgttgtaaaacgacggccagtgagcgcgcgtaatacgactcactatagggcgaattgggtaccCCATCCATGCACACAACCAAATTATATTGCTCTTTATTATGCCTAAATAAGTTGATTTAACATATTACTCTAATAAATAAATTTATATATACATATACAAAATGCTTGTATAAATATATATATATATATATACATATATAGATCTTACCCTTGCCAAAATGTAGATGACGAAGCATAGTGTGATGTCCACCCTCAGCATATCAGATGTGAGCTTACCAAGTGTTGTGTGAAGTCGCAATTTCATAATAAATAAATAATAAGAAGAATTCCAAATTAAAACTTAACTCGCGGATGGAAATGATTCCATCACCAGCACCGTTTCTAGGAACCTTCTTTAAGGCAGTGGTCGGCGAGCGACCTCCAAAGCAAAGCTGATGTACCTATTGACCCCCCCCCCCCCCCCCCCAACCTCCTTGTCTATGTATGTGAACTTGAATCAGGATCCTCACGGTCGTCGCAGTCGTATTTTCACCACTGAGTGCTCATTAGAGTTGGTCGCCAGGATCGGAGCCCTGGGTGCCGCAGTTTGGTGCAACGCTCCGGTAGGCAGGGGTGCTGAAGAATACAACGATCTGGCTGTTGTAGTGAGTTTACTGTCTCTCCCGTTTATTTTTCTTTCGGTATGGAATGGATTTAGAGCGTGGTAATTGTGGGATATGGGTTGTGTTTGTAATGTGTTTTTTTTTTTTTTTTTGAGGGTTGATGTGATTTTGGAGAATTTCAAGGCTTTCTGAGATTGTGGTTTTTGTCGGTGAATTTAGGGTTTGGAGTGGCGACGATTGGGAGACGTCATCGATTGATTTGGCAGTGGGAGTTTGGAGCGGAGGctcgaggtcgacATGGAATATGATTATGGACGAAGCGGGCACGGGAGTGGTGGTTATGAGATGGGAAGGCCGATGTATCATAGTAGACAAGGGAGTAACGTGCAAGGTAGCTATCCCCGAGTAGGGCAATCCGCAGGGGATGCATTGATGAATCGCGGTCCGCCCCAGGCACCTCTTTTATCGGTTCCATCTTTCCCTTCAGGATCAGCTATCAAAGTGACCATCAAACCTATGTATTGATTGGGTCCACCTGCTCAACTAAGAGTGCAGAGCAGGGAGGTGCCCCGGAGCTTATTTCAGTTCGAGTTTGATTTAGAGCGTCGCATCCTTGCAGAGGCGGAGCAGGGAAATCTCAATTTTAGAGCTGGAAGTGGGGCCACTCTAAGTCAATCTAACTCGGAAGCTGATCTTGCTGAAGTGGAAGATGCGACGGTAGCCAAATATCTTGCCATGGGCCATAATAAAGAAGCTGTACAGTATGCTCTTCAAACTTATGGAGACGATCAAAACAAGGTTCTTGATTTCTGTCCTCCTTTCAATAGAATTCGAGAAATGGGGTTTGCAGCCGATCGTGTTGCAAAGGCCTTGGCCAGCTGTAATAATGATGAGGAGCAAGCAATATCATCTCTAGTTTCTaagctttTGGTGAGCAAGGGCGAGGAGCTGTTCACCGGGGTGGTGCCCATCCTGGTCGAGCTGGACGGCGACGTAAACGGCCACAAGTTCAGCGTGTCCGGCGAGGGCGAGGGCGATGCCACCTACGGCAAGCTGACCCTGAAGTTCATCTGCACCACCGGCAAGCTGCCCGTGCCCTGGCCCACCCTCGTGACCACCTTCACCTACGGCGTGCAGTGCTTCAGCCGCTACCCCGACCACATGAAGCAGCACGACTTCTTCAAGTCCGCCATGCCCGAAGGCTACGTCCAGGAGCGCACCATCTTCTTCAAGGACGACGGCAACTACAAGACCCGCGCCGAGGTGAAGTTCGAGGGCGACACCCTGGTGAACCGCATCGAGCTGAAGGGCATCGACTTCAAGGAGGACGGCAACATCCTGGGGCACAAGCTGGAGTACAACTACAACAGCCACAACGTCTATATCATGGCCGACAAGCAGAAGAACGGCATCAAGGTGAACTTCAAGATCCGCCACAACATCGAGGACGGCAGCGTGCAGCTCGCCGACCACTACCAGCAGAACACCCCCATCGGCGACGGCCCCGTGCTGCTGCCCGACAACCACTACCTGAGCACCCAGTCCGCCCTGAGCAAAGACCCCAACGAGAAGCGCGATCACATGGTCCTGCTGGAGTTCGTGACCGCCGCCGGGATCACTCACGGCATGGACGAGCTGTACAAGtaaTCGACCGCTCCCGCCATCGATGATCGTTCAAACATTTGGCAATAAAGTTTCTTAAGATTGAATCCTGTTGCCGGTCTTGCGATGATTATCATATAATTTCTGTTGAATTACGTTAAGCATGTAATAATTAACATGTAATGCATGACGTTATTTATGAGATGGGTTTTTATGATTAGAGTCCCGCAATTATACATTTAATACGCGATAGAAAACAAAATATAGCGCGCAAACTAGGATAAATTATCGCGCGCGGTGTCATCTATGTTACTAGATCCGATGATAAGCTGTCAAACATGAGAATTCctgcagcccGGTACCCCTACTCCAAAAATGTCAAAGATACAGTCTCAGAAGACCAAAGGGCTATTGAGACTTTTCAACAAAGGGTAATTTCGGGAAACCTCCTCGGATTCCATTGCCCAGCTATCTGTCACTTCATCGAAAGGACAGTAGAAAAGGAAGGTGGCTCCTACAAATGCCATCATTGCGATAAAGGAAAGGCTATCATTCAAGATGCCTCTGCCGACAGTGGTCCCAAAGATGGACCCCCACCCNCGAGGAGCATCGTGGAAAAAGAAGACGTTCCAACCACGTCTTCAAAGCAAGTGGATTGATGTGACATCTCCACTGACGTAAGGGATGACGCACAATCCCACTATCCTTCGCAAGACCCTTCCTCTATATAAGGAAGTTCATTTCATTTGGAGAGGACAGCCCAAGCATGGAGAACCGACCTGCAGGCATGATTGAACAAGATGGATTGCACGCAGGTTCTCCGGCCGCTTGGGTGGAGAGGCTATTCGGCTATGACTGGGCACAACAGACAATCGGCTGCTCTGATGCCGCCGTGTTCCGGCTGTCAGCGCAGGGGCGCCCGGTTCTTTTTGTCAAGACCGACCTGTCCGGTGCCCTGAATGAACTCCAAGACGAGGCAGCGCGGCTATCGTGGCTGGCCACGACGGGCGTTCCTTGCGCAGCTGTGCTCGACGTTGTCACTGAAGCGGGAAGGGACTGGCTGCTATTGGGCGAAGTGCCGGGGCAGGATCTCCTGTCATCTCACCTTGCTCCTGCCGAGAAAGTATCCATCATGGCTGATGCAATGCGGCGGCTGCATACGCTTGATCCGGCTACCTGCCCATTCGACCACCAAGCGAAACATCGCATCGAGCGAGCACGTACTCGGATGGAAGCCGGTCTTGTCGATCAGGATGATCTGGACGAAGAGCATCAGGGGCTCGCGCCAGCCGAACTGTTCGCCAGGCTCAAGGCGCGGATGCCCGACGGCGAGGATCTCGTCGTGACCCATGGCGATGCCTGCTTGCCGAATATCATGGTGGAAAATGGCCGCTTTTCTGGATTCATCGACTGTGGCCGGCTGGGTGTGGCGGACCGCTATCAGGACATAGCGTTGGCTACCCGTGATATTGCTGAAGAGCTTGGCGGCGAATGGGCTGACCGCTTCCTCGTGCTTTACGGTATCGCCGCTCCCGATTCGCAGCGCATCGCCTTCTATCGCCTTCTTGACGAGTTCTTCTGAGCGGGACTCTGGGGTTCGGACGGTACGCTGAAATCACCAGTCTCTTCTACAAATCTATCTCTCTCTATTTTCTCCATAAATAATGTGTGAGTAGTTTCCCGATAAGGGAAATTAGGGTTCTTATAGGGTTTCGCTCATGTGTTGAGCATATAAGAAACCCTTAGTATGTATTTGTATTTGTAAAATACTTCTATCAATAAAATTTCTAATTCCTAAAACCAAAATCCAGTACTAAAATCCAGATCTCCTAAAGTCCCTATAGATCTTTGTCGTGAATATAAACCAGACACGAGACGACTAAACCTGGAGCCCAGACGCCGTTCGAAGCTAGAAGTACCGCTTAGGCAGGAGGCCGTTAGGGAAAAGATGCTAAGGCAGGGTTGGTTACGTTGACTCCCCCGTAGGTTTGGTTTAAATATGATGAAGTGGACGGAAGGAAGGAGGAAGACAAGGAAGGATAAGGTTGCAGGCCCTGTGCAAGGTAAGAAGATGGAAATTTGATAGAGGTACGCTACTATACTTATACTATACGCTAAGGGAATGCTTGTATTTATACCCTATACCCCCTAATAACCCCTTATCAATTTAAGAAATAATCCGCATAAGCCCCCGCTTAAAAATTGGTATCAGAGCCATGAATAGGTCTATGACCAAAACTCAAGAGGATAAAACCTCACCAAAATACGAAAGAGTTCTTAACTCTAAAGATAAAAGATCTTTCAAGATCAAAACTAGTTCCCTCACACCGGTGACGGGGATCGCATGCGATATCTCGAGgggggatccactagttctagagcggccgcTAATCTGTGTATGAGTTCAGGTGAGATCTCTTGAAGATCCTTGGAGCATTCTGTCGCACTCGTAACAAGTTCTCGGATTGTCTCGGAGGTGGGCTTTTCAACTTGCAGGCCTTAAGTCAAGGTCTTCTTAGAAGCGACTTTCCTGCACATGTTAATCCAATGATAAAACACTTCAAGAAACTCATCTTTTCTTGATGTGAGTAACTATTAGTTTCATTAGGATTACCTTTGCAATTAGCGCAGTTCGGACGATCTCAGTTTTGGACACACATAGGTCATATTTCCAGAGAGCTCATTCGGTAGCAGCGTTACCCATAATGAATCAGGTTGCAGAAATTCGTGGAGCATTAAGTGGTACTTTCATGGGAAGTATGTACATTGGAGATGGTTATATTCCTTAGGTATTCAGCGTAATAACAAGTCTTAGAGATTACACATCTTTGCCACAGGCTAAGCACTTAGTTCTCAATCTTGTGAAATATAGTACAAGTATCCACTATTGACTGTCTCCCCTTGTCTTCACTGTTGACCTTATACTCGACTCATTTTTGAATTATGAGATTGTATTATGACCTATCATCATGGTATCGAAAGCGTCCTATCTTCATGCCTCGTATGCTCCAATGCAATGAAAGTCTTGAAGCTATGGCTAGTTATTTTGTCAAGTCTACCGTCTACCGAATCTAGACGAATACTTTTAGCTCTACTTCAGCAGATTTTCTTTTCTCTAAGCATGTGAATGAGGGCTGCTCCCTTTTAGTTTTCGGGAGAAGTTCCTTATCAAAGGCTTATTATCAGGCAAAAAGAGATGGATATAAAGGTTTTGTAGTGCCTATGATTGTCACCATCAAGTCATGATGCTTGTATGCAGAATTTTTGTTAATGGGCAGTCTATTGCATCATGGCTTTTGTTCTGGCTTGCATTTGGACATGAATGGTTTTTTTTTTAGTGTATTGGATCTATGCTCTTGAGAAAATATCAAGCTGATGAACTCAACTACTTACCATTTCAAGTTTCCAGTTAGTGATCAGTGATGCTACAGTGACTTTTGTCTTTAGTTTTCTGGTTGTTTCGAGTGTCTTGAACAAGATGATAGAGGTTTCTGTGGAATTGGAGACCTCATAGGACGTTCAAGATGTGGAGGGGAATCTCTTAAAACCTAAACATGACAAATGAAATAGGACTAGTAATTAAGAGAATTAATTGATCAAGTTCAAAATCTACATAAACTTCAAATTTATGCACTATATTACCTTAACTGGTAGATCAAAACTTATTCTTATAATTTGAATTCTCATGTCATAAGGAAGTCAGAACTTTTGATTTCCAGGATTTCTATAAACTTCAACTGAAGTAAATGTTGAAGTAGCAAATGTACCTCCATACAATCTGATTAACCTCCACAAAACTAGATAACTCccgcggtggagctccagcttttgttccctttagtgagggttaattgcgcgcttggcgtaatcatggtcatagctgtttcctgtgtgaaattgttatccgctcacaattccacacaacatacgagccggaagcataaagtgtaaagcctggggtgcctaatgagtgagctaactcacattaattgcgttgcgctcactgcccgctttccagtcgggaaacctgtcgtgccagctgcattaatgaatcggccaacgcgcggggagaggcggtttgcgtattgggcgctcttccgcttcctcgctcactgactcgctgcgctcggtcgttcggctgcggcgagcggtatcagctcactcaaaggcggtaatacggttatccacagaatcaggggataacgcaggaaagaacatgtgagcaaaaggccagcaaaaggccaggaaccgtaaaaaggccgcgttgctggcgtttttccataggctccgcccccctgacgagcatcacaaaaatcgacgctcaagtcagaggtggcgaaacccgacaggactataaagataccaggcgtttccccctggaagctccctcgtgcgctctcctgttccgaccctgccgcttaccggatacctgtccgcctttctcccttcgggaagcgtggcgctttctcatagctcacgctgtaggtatctcagttcggtgtaggtcgttcgctccaagctgggctgtgtgcacgaaccccccgttcagcccgaccgctgcgccttatccggtaactatcgtcttgagtccaacccggtaagacacgacttatcgccactggcagcagccactggtaacaggattagcagagcgaggtatgtaggcggtgctacagagttcttgaagtggtggcctaactacggctacactagaaggacagtatttggtatctgcgctctgctgaagccagttaccttcggaaaaagagttggtagctcttgatccggcaaacaaaccaccgctggtagcggtggtttttttgtttgcaagcagcagattacgcgcagaaaaaaaggatctcaagaagatcctttgatcttttctacggggtctgacgctcagtggaacgaaaactcacgttaagggattttggtcatgagattatcaaaaaggatcttcacctagatccttttaaattaaaaatgaagttttaaatcaatctaaagtatatatgagtaaacttggtctgacagttaccaatgcttaatcagtgaggcacctatctcagcgatctgtctatttcgttcatccatagttgcctgactccccgtcgtgtagataactacgatacgggagggcttaccatctggccccagtgctgcaatgataccgcgagacccacgctcaccggctccagatttatcagcaataaaccagccagccggaagggccgagcgcagaagtggtcctgcaactttatccgcctccatccagtctattaattgttgccgggaagctagagtaagtagttcgccagttaatagtttgcgcaacgttgttgccattgctacaggcatcgtggtgtcacgctcgtcgtttggtatggcttcattcagctccggttcccaacgatcaaggcgagttacatgatcccccatgttgtgcaaaaaagcggttagctccttcggtcctccgatcgttgtcagaagtaagttggccgcagtgttatcactcatggttatggcagcactgcataattctcttactgtcatgccatccgtaagatgcttttctgtgactggtgagtactcaaccaagtcattctgagaatagtgtatgcggcgaccgagttgctcttgcccggcgtcaatacgggataataccgcgccacatagcagaactttaaaagtgctcatcattggaaaacgttcttcggggcgaaaactctcaaggatcttaccgctgttgagatccagttcgatgtaacccactcgtgcacccaactgatcttcagcatcttttactttcaccagcgtttctgggtgagcaaaaacaggaaggcaaaatgccgcaaaaaagggaataagggcgacacggaaatgttgaatactcatactcttcctttttcaatattattgaagcatttatcagggttattgtctcatgagcggatacatatttgaatgtatttagaaaaataaacaaataggggttccgcgcacatttccccgaaaagtgccac
