# Supplementary material for: Genetic Regulation of the 2D to 3D Growth Transition in the Moss Physcomitrella patens
Source: Curr Biol. 2018 Feb 5;28(3):473–478.e5. doi: 10.1016/j.cub.2017.12.052 (PMC5807088; doi:10.1016/j.cub.2017.12.052)
Supplement: Data S3. NOG1 Protein Sequences [file mmc4.docx]

**DATA S3.**

**A) COMPLETE SET**

>P.patens_PhPat.001G046300.1_NOG1

MEYDYGRSGHGSGGYEMGRPMYHSRQGSNVQGSYPRVGQSAGDALMNRGPPQAPLLSVPSFPSGSAIKVTIKPMYRLGPPAQLRVQSREVPRSLFQFEFDLERRILAEAEQGNLNFRAGSGATLSQSNSEADLAEVEDATVAKYLAMGHNKEAVQYALQTYGDDQNKVLDFCPPFNRIREMGFAADRVAKALASCNNDEEQAISSLVS

>M.polymorpha_0050s0076.1
MDFDYRKGGGGLTTSGRQQASGYDSNSRQPMYVPRYERSGSALVSQNQAYYPRIAQPVGPSVNRPPAPIPNNPSASNLPGSGIRVAIKPEYRVGPAVQLILPQNAEVPRSLFNFDFELERRILAEAERGAQGWDPARANGEQNLTPGMVDDPVVRKYLAMGLHREAVVMAVTTYGDVQNKVLDFVPSYNLLREMGFPAVAVTGALAMYDNDRERALAHFV

>S.fallax_0183s0013.1
MEQEYRKGGQTTRTQSSSGGGYDARPLYRMMTTSSQVNTDSSSSSFYPRIAQPVGGPPVNRPPPPPIAASNSFSSGTGIKVTIKPIYRVAPPVQLLPQGGEVPRSLFQFDFDLERRILAEAESQGQSFSRKPVDHSRQSNGGSVEDPTVAKYVSMGLNKEAVVMAIRTFGDIQNKVVDFCPTYSRLWEMGFQSDAVAAALATCDNDFERALAYLVSM

>S.fallax_0069s0038.1
MEYDYRKGGGGQSRSQSSSSGYDAARYNRLQSNNSSSSLYPRIAQPFGPSVNRPPPPPTIATSSSSSSGTGIKVTIKPIYQVAPPVQLLPQGGEVPRSLFQFDFDLERRILAAGESPGQPSYSTICADHSRLANGASASLAEVDDPAVGKYIAMGLNKEAVVMAIRTLGDVQNKVLDFCPSYDRLQEMGFQPDAVAAALAKSDIDFEQSLARLVSL

>S.moellendorfii_predicted

MEAAYDRRRYDQHHPTRPPPPNPSSSSFYPRLPQQAPAPPTVNRAALPPVPGAPPQSSGLGIKVALKPQYRMAPPVQLSPVIGEVPRTSIAQFDFDFERKVIDDAERGVRTQIRQHQLANGGETASASQANDDPVISKYLSMGLSREAVSLGVAAYGDVQAKVVEFCSAYSLLREMGFPSTAVAGALVMHDNDKEKALAQFV

>A.trichopoda_evm_27.model.AmTr_v1.0_scaffold00001.468
MDYDFRKGASPYDSSSAIPMYRPPSSYGAHGSPSYYPAPNRIAHTSGPPVSRPPPVPTTSSSPSSSLGIRVAIKPAYRITPPPQLSPQMAEIPRCSFLFDFEFERKILAEAEKESQNWSKIATETPSSSKPTESGTQESAADQLVNKYAALGLNREAVSFAVANYGDNANKVREFVSSYNLLREMGFASTNVARVLVMYDNDRDKALAHFLGSSV

>Z.marina_Zosma267g00330.1
MDYSYRHRNSYSPRAPPPPITSSMYPKMGSQPIQTDHAPPLPVRGSFAPQHNPPPPSSSTTGMGIRVSIKPEYRITSPPELFPQAKEVPQSKFHFDFEFERKFLAEIEKNGFCNWSKFSSEYQPLKDHTSSNSSMGAVNDVVVDKYTSSGLGREAVSFAVLNYGDNPVKVREFVKSYNLLREMGFVSKDVAEVLAMYDNDTDKALAHLLNTVKG

>S.polyrhiza_26G0010900
MDYDYRNRTGPVNRPAPTSSIYPRVSQQPGQHVPVPAGRATHPHPAAAPPTSGSGIIRVMLKPEYRITPPPQLAPQVTEVPRSHFNFDFEFERRILAEAEKDGQNWTRIVSENQSSFSASASSAGPAGDPVVEKYVAMGLGREAASLAVLSFGDDAIKVREFVKGYNLLREMGFASKNVAEALVMYDNDTDRALAHFLNSSS

>S.bicolor_Sobic.010G047400.1
MEYDFRGSRSGSGPGPYGAPPGAAPGGGSSLYPRVGQPSHGGGGASTASPRAAPYHHGSGSGSSAPVVTPLAPISSSSSSKVGIQVTIKPEFRIDPPPQLPPQMVEIPRSTFNFDFEYERRILAEAEKENPNWTKFVVERQAPPPVAQQARPASSGDPVVDRYVSMGLGREAVSFAVLNYGDNPTKVKEFVKSYNILHEMGFTSPNVPELLAIHDNDPDKVIQRLLSSPS

>S.viridis_4G063700.1
MEYDFRGRPGSGSYGAPPGAAAPGGGSSLYPRVGQPTHGGGGGGSATASPRAAPYHHGPGAGSGSSAPIVTPLAPTSTSSKVGIQVAIKPEFRITPPPQLPPQMVEIPRSTFNFDFEYERRILAEAEKENPNWSKFVVERQAPPPPVPQQARPASSGSGDPVVDKYVSMGLGREAVSFAVLNYGDNPAKVKEFVKSYNILHEMGFTSPNVPELLAIHDNDPDKVIQRLLSSPS

>S.italica_4G065800.2
MEYDFRGRPGSGSYGAPPGAAAPGGGSSLYPRVGQPTHGGGGGGGGSATASPRAAPYHHGPGAGSGSSAPIVTPLAPTSTSSKVGIQVAIKPEFRITPPPQLPPQMVEIPRSTFNFDFEYERRILAEAEKENPNWSKFVVERQAPPPPVPQQARPASSGSGDPVVDKYVSMGLGREAVSFAVLNYGDNPAKMYLSSAGIISVWQLSKFKLQKIIYSEHTSCFFKGEGVCEIVQHPPRDGLHIAKRPRAAGDPRQRP

>B.distachyon_1g48630.2
MEYDFRGRPGSGSGSYAGSSGGGSSLYPRVGQPSHGGGSAPLQRPAPYLHPSAVASPAPNAPAPASSSSTSMGIQVVIKPEYRITPPPQLTPQMVEVSRSTFNFDFEYERKILAEAEKENPNWSKFVVERQTPPPPQPQPPRGPRHTTSTTSMGMQATPGDPVVQKYISMGLGREAVSFAVLNYGDNPTKVKEFVKSYNALHEMGFTSSNVPELLAMHDNDPDKVIQHLLSTP

>B.stacei_07G040400.1
MEYDFRGRPGSGSGSGSGSYAGSSGGGSSLYPRVGQPSHGGGGAPPQRPAPYLHASAVPSPAPNAPAPAPSSSTSMGIQVVIKPEYRITPPPQLTPQMVEVPHSTFNFDFEYERKILAEAEKENPNWSKFVVERQTPPPPQPQPPRGPRHTTSTTSMATPGDPVVQKYISMGLGREAVSFAVLNYGDNPTKVKEFVKSYNALHEMGFTSSNVPELLAIHDNDPDKVIQHLLSTP

>O.sativa_LOC_Os06g06530.1
MDYDYRGRPGSGSYGGGVGGGGGSSSLYPRVGQPSHGVANAPPPEPPRAAPYHHHGPPTVSAAPHPVPASSSTSMGIQVVIKPAYRITPPPQLPPQLTEIPRSTFNFDFEYERKILAEAEKENPNWSKFVIESQPPPPPQPPRGPKLTTPTTSVATPGDPVVDKYISMGLGREAVSFAVLNYGDNPAKVKEFVKSYNALHEMGFTSSNVPELLAIHDNDPDKVIQHLIGTS

>Z.mays_GRMZM2G071249_T01
MEYDFRGGRSSSGPGPYGAPPGGAPGGGSSLYPRVGQPSHGGGGASTASPRAAPYHHGSGSGSGSGSSAPVVTPLAPTSFSSSSSSSSKVGIQVAIKPEFRITPPPQLPPEMVEIPRSTFNFDFEYERMILAEAEKENPNWTKFVVERQAAPLVPQQARPASSGSGDPVVDKYVSMGLGRQAVSFAVLNYGDNPTKVKEFVKSYSILHEMGFTSPNVPELLAIHDNDPDKVIQRLLSSPS

>P.hallii_J00097.1
MEYDYRGRPGSGSYGAAPGGGGGGPSLYPHVGQPSHGGGGSGTASPRAAPYHHGPGGGSGSSAPIVTPLAPTSTSSSKVGIHVAIKPEFRITPPPQLPPQMVEIPRSTFNFDFDYERRILAEAEKENPNWSKFVVERQAPPPVPQQQARPASSGSGDPVVDKYVAMGLGREAVSFAVLNYGDNPAKVKEFVKSYNILHEMGFTSPNVPELLAIHDNDPDKVIQRLLSSPS

>P.virgatum_Da02161.1
MEYDFRGRPGSGSYGAPPGAAPGGGGGGTSLYPRVGQPSHGGGGAGTASPRAAPYHHGHGSGSGSSAPIVTPLAPTSSSSSSKVGIHIAIKPEFRITPPPQLPPQMVEIPRSTFNFDFEYERRILAEAEKENPNWSKFVVERQAPLPVPQQQARPASSGSGDPVVDKYVAMGLGREAVSFAVLNYGDNPAKVKEFVKSYNILHEMGFTSPNVPELLAIHDNDPDKVIQRLLSSPS

>P.virgatum_J06390.1
TASPRAAPYHHGPGGSSGSSAPIVTPLAPTSTSSSKVGIHVAIKPEFRITPPPQLPPQMVEIPRSTFNFDFEYERRILAEAEKENPNWSKFVVERQAPLPVPQQQARPASSGSGDPVVDKYVAMGLGREAVSFAVLNYGDNPAKVKEFVKSYNILQREWASHRK

>A.corulea_5G443200.1
MDYDFRNKATPYDTHIPMYRPTTTTSSSPSPHISSFYPKVGQQQQQPPLRSSSFHQNLPPPPPPSSSSSSAGMGIRVTIKPEYRINPPPQLSPHVGEIPRITFQFDFDFERKVLAEAEKETPNWSRLGLDNGQLRTAEPISSPGPVRDPVVSKYIASGLSREAVPLAVANYGDNPSKDVIYLQVQEFVNGYNLLREMGFSSSKVAEALAMYDNDTDKALAYFLNNSS

>S.lycopersicon_03g007520.2.1
MEYDFRNRTGPPYDTQSPMYGRPATGAPQPHPMYGQAPGLYPRPGQHSGGRNPPFHHTPPPSSNTGIGIRVAIKPEYRITPPPQLSTQVGEIPRSTFNFDFDLEKKILAEAEKESQNWSKLGLENLPPIMPEQPSMGYTGDLMMNKYATAGFNREAVAIAVANYGDNPIKVKEFVEGYTLLKEMGFSSNSVADALLMNDNDKDKAAAQLLGNSS

>S.lycopersicon_06g036500.1.1
MEPSYSTQSPMYSRPATTALPLTIHHSPMYGQASAEIGIRIAIIPEFQITPPPPLVPQGDVPQCTFQFDFDLEKKILAEANQKESQIWSRLPIENLPSRGSDQASKVHFHVNL

>S.tuberosum_PGSC0003DMT400023416
MEYDFRNRTGPPYDTQLPMYGRPATGAPHPHPNPQPHPMYGQAPGLYPRPGQHSGVRNPPFHHIPPPSSNTGIGIRVAIKPEYRITPPPQLSTQVGEIPRSTFNFDFDLEKKILAEAEKESQNWSRLGLENLPPRMPEQTNMGYTGDPMMNKYATSGFNREAVAIAVANYGDNPIKVKEFVEGYTLLKEMGFSSNSVADALMMNDNDKDKAAAQLLGNSS

>S.tuberosum_PGSC0003DMT400074355
MAYDLRNRTDPSYGTQSPKYGRPATTAPPLPSHHHPMYGQPSSLYPRVGQHSGGNFPGRFNPTPSPSSTTGIGIRVAIKPEYQITPPPPLAPQGDVPQCTFQFDFDLEKKILAEADQKESQIWSRLPLENLPSRGSDSDQASKASSGDPVMIKYIAFGLNREAVPLAVANYGDNPNKVTEFADGYTYLKEMGFSSNSAADALLMNDNDTNKAIQHLLNNPSY

>M.guttatus_F00251.1
MEYDFRKRTGSTYDSNVPSYSRPPPTASSTGAAQPMYGQSPSIYPKISGAGSHSSASIVRNPPFHQAPPPPSTSGMGIRIAIKPEYRITPPPPMFPQTGEIPRSNFHFDFEFEKNVLTELVKEKPNLGWIVNENPPPKATESTSSYGPSADPIVSKYIATGLHVEAVRLAVANYGDNPTKVKEFANGYNILQEMGFSSNNVAEALFMYDNDTDKALAHFLNATS

>E.grandis_A02672.2
MYPRIGSHHGNPAVTAGRASSFHHAAPPASGAGLGIRVAVKPEYRITPPPQLIQQMGDIPRSNFQFDFDFERKVLAELEKESPDWSRLGLENLPSRSSEAPPPSGPAGDPIINKYISSGLSREAVPLAVANYGDNPTKVREFVNGYTRLREMGFSSSSIAEALVKNNNNMDEALAYFLNSSS

>R.communis_30169.m006549
MDYDFRNRTTGSSYDSQTPMYRTTSSSTPSGHPMYGQSLYPRIGQQQGHTPVPPVGRHSSYHQSSAPPPSSSGLGIRVSVKPEYRITPPPQLAPQMGDIPRSNFQFHFDLERRILAEAEKDIQNWSKLGLENLPSKTTESTSSLGSAADPVVSKYIASGLNREAVPLAVANYGDNPTKVREFVNGFSLLREMGFSANNVAEALLMYDNDTDKALTHFLNSSS

>P.trichocarpa_ 015G023600.1
MDYDFRNRTSSPYDTPSTMHRSSTPSTAPQPSHPMYGPPSLYPTVNQPGHTVIPHAPRHHSFTQQAPSSPSSGLGIRVMIKPEYRITPPPQLTPQIVEIPRSSFQFDFELERQILAEAEKDSPNWSRLLGLENSPPKPPPILGSSLIVGKYPVVRKYISMGLNRDAVPLAVANYGDNPPKVQEFVNGYTLLQEMGFPSNKVAEALLMYDNNTDEALAHFLNSS

>P.trichocarpa_012G033300.1
MEYDFRNRTSSPYDTQSPMYRSSTPLTTAPPPTHPMYGPSLYPRVSQPAHPAIPPVSRHHSFPQPSSSSPSSGLGIRVMIKPEYRITPPPQLTPQIGEIPRSSVQFDFELERQIIAEAEKGSVNWSRLLGLENLPSKPLESTPSTGPTADPVERKYIASGLSRDAVPLAVANYGDNPTKVQEFVNGYTLLREMGFSSSSVAEALLTYDNDADKALAHFLGSS

>M.esculenta_14G019500.1
MDYDFRNRAASPYDQIPIYRTSSSSSAPSSHPMYGQSLYPRIGQQGHAAVPPSGRHSSYHQTSAPSPSSSGLGIRVAVKPEYRITPPPQLSPQVGDIPRSSFQFDFEFERKILAEAEKESQNWSKLGLENLPSKTTESTSSLGSTVDPVVSKYIRSGLKPEAVPFAVANYGDNPTKVQEFVNGYSLLREMGFASNSVAEALLMYDNDTDKALAHFLNNSS

>M.esculenta_06G156000.1
MYRTSSSSSAPSSHPMYGHSFYPRIGQQEQTAVPPVPRNSLYHQTSAPSPSSSGLGIRVALKPEYRITPLPQLSPQLGDIPRSNFQFDFEFERKVLAEAEKETPSWSKLGLESLPSKTTESTSSLGPTVDPVVSRYVASGLNREAVSLAVANYGDNPTKVQAFVNGYTLLREMGFSSNKVTEALLMYDNDTDKALAHFLNSSS

>L.ussitassimum_10038829
MDYDYRNRSNSPYDPQIPNYNRSPTSSSSAHPMYGAPAYPRVGGGYGGAPPVGRHPSYHQNSAPPPSSSSASGLGIRVALKPEYRISPPPQLLPMREVPRSNFQFDFEFERKLLAEAEKGEINWSRLGMDNLPPKATESTSSSSGSGGDPVVSKYIAAGLNREAVPVAVANYGDNPTKVQEFAKGYTLLREMGFSSNNVAEALLMNDNDTDKALAHFLNSPS

> L.ussitassimum_10014944
MDYDFRNRSNSPYDPQIPSYNRSSTSSSSAHPMYGAPAYPRVGGGYGGAPPVGRHPSYHQNSAPPPSSSSSGLGIRVALKPEYRISPPPQLLPMREVPRSNFQFDFEFERKLLAEAEKGEINWSRLGLDNLPPKATESTSSSSGSGGDPVVSKYIAAGLNRDAVPVAVANYGDNPAKVQEFAKGYTLLREMGFSSNNVAEALLMNDNDTDKALAHFLNSPS

>G.raimondii_007G014400.1
MDYDFRNRTGPPYEAQIPIYRQQPTSSSSTHPMYGSSMYPRIGGQPAAHSVAPPTTRASSFHQNFSPSSSSGLGIRVALKPKYRITPPPQLSPQVGDIPRSNFQFDFEFERKILAEAEKENMNWSKLGLENLSSKPIETSSSSTGANSDPVVSKYIASGLSREAVTLAVANYGDNPTKVREFVTGYNLLREMGFSSNNVAEALLMYDNDTDKALAHFLNSSS

> G.raimondii_006G200100.1
MYRQQLTSTSSSSSRPMYRPSIYPNVGQPGHPVVPPAPRTSSSPSSSAGLGIRVVLKPDYRITPLPQFSPQVGDIRRSNFQFDFEFERKILAQPDAEFMNLSQLDLENHPSEPTQSTPSSGANSDSVLNKYIASGLSQEAVIIAVANYGDSPTKVREFVNGYNLLREMGFSANNVADALLVCDNDTDKALAHFLNSSS

>C.sinensus_orange1.1g046691m
MDYDFRNRANSSYSLYGPPPSASSGGSAPSHHPMYGSSLYPRIGQQGSGHSMGPPVSRTSSYNASPSPSSSSSGLGIRVALKPEYRITPPPTLSPQVGDIPRSNFHFDFDFERRVLAEAEKENQNWSRLGMENIPSKNEPTSSVGSGSDPVVSRYIALGLNREAVHIAVANYGDNPTKVREFANGYTILREMGFSSNNVAEVLIMYENDTDKALAHLLGSSA

>T.cacao_1EG015327t1
MDYDFRNRTGPPYEAQIPMYRQQPTSSSSSSSMPSSHPMYGPSLYPRIGQPAHTVVPPAPRIPSFHHTSSPSSSSGLGVRVALKPEYRITPPPQLLPQVGDIPRSNFQFDFGFERKILAEAENESMNFSRLGLESLSKSTESTSSSGANADPVVSKYIASGLNREAVTVAVANYGDNPTKVREFVHSYNLLREMGFSSNNVAEALLMYENDTDKALAHFLNNSS

>A.thaliana_AT5G53330.1
MDYDYRNKSGGPSYPRPMYGPPSTSPSPSSNHPMYGYPKIGQQTGPGPQFFSPPERNSSFQHNTSPSSGIGIRVNLKPEYRITPPPQLLPRVGDIHRSSFQFDFGLERKVLAEAEKDNPDWSKFGSENPPAKFHEPSPSSVGQMQGVDHVVMKYTASGLNREAVNIAVANYGDNPTKVQEFANGFTAIREMGFPTNAVADALFMFENDTDKALAHLLHGSS

>B.rapa_J00835.1
MDYDYRNKSGPSYARPMYGYPNIGQQSGHGHQFFPPPERNQSFQQHNSSPFPFSSSSSSSGLGIKVTLKPEFRITPPPQLLPRAGDIPRSGFQFDFGLERAVLTEAEKDNPDWSKFGSDIPPPSNFPQPPPVPSMGVDPLVMKYTASGLNREAVNIAVANYGDNPTKVQEFANGFAAMREMGFPTNAVAEALFMFDNDTDKALSHLLHGSS

>B.rapa_C01396.1
MDYDFRNKSGPPYGRPMYPSPSPSSTHPMYNGPPGYPKIGQQSSHGQPFFPPPERNPSFQHNPSPSSSSGLGIKVTLKPEYRITPPPPLLPRVGDVPRSSFQFDFGLERKILAEAEKENPDWSKFGSEHPPPPSNFPPTPSPSMGVDPVVMKYAGLNREAVNIAVANYGDNPTKVQEFANGFTALREMGFPTNAVADALFMFENDTEKALSHLLHGSS

>B.oleracea_030304
MDYDYRNKFGASYARPMYGPPSISPSPPSTHPMYGYPNIGQQSGHGHQFFPPPERNQSFQHNSSPSPFSSSSGLGIKVTLKPEYRITPPPQLLPRAGDIPRSGFQFDFCLERAVLAEAEKDNPDWSKFGSDIPPPSNFPQPPPVPSMGVDPLVMKYTASGLNREAVNIAVANYGDNPTKVQEFANGFAAMREMGFPTNAVAEALFMFDNDTDKALSHLLHGSS

>B.oleracea_027997
MDYDFRNKSGPPYGRPMYPSPSPSSTHPMYNGPPGYPKIGQQSSHGQPFFPPPERNPSFQHNPSPSSSSGLGIKVALKPEYRITPPPPLLPRAGDIPRSSFQFDFGLERKVLAEAEKENPDWSKFGSEHPPPPSSFPPTPSPSMGVDPVVMKYAGLNREAVNIAVANYGDNPTKVQEFANGFTALREMGFPTNAVSDALFMFENDTEKALSHLLHGSS

>C.rubella_10027076m
MDYDYRNKSGPSYPRPMYEPSSASSPSPASNHPMYAPSGYPKIGQQTGHGQQFFPPPERNSSFQHNTPPPFPSSSSSSGLGIKVNLKPEYRITPPPQLLPRVGDIHRSSFQFDFGLERKVLAEAEKDTPDWSKFGTENPPAKFPEPSSSSMGVDPVMKYAGLNREAVNIAVANYGDNPTKVQEFANGFTAIREMGFPPNSVAEALFMFENDTEKALAHLLHGSS

>C.grandiflora_0084s0078.1
MDYDYRNKSGPSYPRPMYEPSSASSPSPASNHPMYAPSGYPKIGQQTGHGQQFFPPPERNSSFQHNNPPPFPSSSSSSGLGIKVNLKPEYRITPPPQLLPRVGDIHRSSFQFDFGLERKVLAEAEKDTPDWSKFGTENPPAKFPEPSSSSVGVDPVMKYAGLNREAVNIAVANYGDNPTKVQEFANGFTAIREMGFPTNSVAEALFMFENDTEKALAHLLHGSS

>E.salsugineum_10014603m
MDYDFRNKSGPPYARPMYGTASASPSPPSSHSMYGPPGYPKIGQQSGHGQLFFPPPERNSSFQHNSSPFPSSSSSGLGIKVTMKPEYRITPPPQLLPRVADIPRSSFQFDFSLEKKVLAEAEKENPDWSKFGTENPPQAKFPEPAPSMGVDPIVMKYAGSGLNREAVNIAVANYGDNPTKVQEFANGFSAIREMGFPTNAVADALFMFENDTEKALAHLLHGSS

>A.lyrata_AL8G28040.t1
MDYDYRNKSGPSYPRPMYGPPSTSPSPASNHPMYGYPKIGQQTGHGQQFFPPPERNSSFQHNTSPSSGLGIKVNLKPEYRITPPPQLLPRVGDIHRSSFQFDFGLERKVLAEAEKDNPDWSKFGSDNPPAKFLEPTSSSVGVDPVVMKYAASGLNPEAVNIAVANYGDNPTKVQEFANGFTAIREMGFPTNSVADALFMFENDTEKALAHLLHGSS

>C.sativus_201900.1
MAYDFRNNSGHYDSHQPMYTSTASSSPSPSPHPMYSHSMYPRIGQQAPSSTPPVARLSSHHYSSSPSPSPSSSCKFIAGLGIRVTIKPEYRITPPPQLSPQVGDIPRSNFQFDFEFEKKVLAEAEKEAPNWNRFGLEHPPPKPVESTSSMGSIGDPVVSKYVASGLNREAVSFAVANYGDNPTKVQEFVKGYTLLREMGFSSIKVVEALLMYDNDTDKAVAHFLGGTS

>M.trunculata_3g090640.1
MEYEYNRSRSGPQVPMYRAPPSIYPKIGPHPHSAAPRPPPFQHQNPNPSPSIGLGIKVAIKPEYKIAPPPHLLPHVGDIPRSNFQFDFGLERKILAEAEKENPNWTKFGVENLPTKASDSSPSSKVTTADPIVNKYIAMGLSRDVVPIAVKNYGDNPTKVQEFVKGYTLLHEMGFSSNSVDEALLMYDNDTDKALAYFLNGSS

>P.persica_2G306000.1
MDYDYRTRSGSPYDSHVYRPATSSAPSSHPMYGPPSSSSMYPRVGQQGQTAAPPPYAHSGRPLPHHQTTTPSSSSSSGLGIRVTIKPEYRITPPPTFSFQVGDIPRSSFQFDFDFERKVLAELEKETQNWAKLGLENPPQRPVESPSSSGSVADPIVSKYIASGLSREAVPLAVANYGDNPTKVREFAKSFTQLREMGFASNDVAEALIMYENDTDKAVAHFLNSSS

>G.max_06G124600.1
MDYDFRSRSGPQPPMYRPPPPPSPMYRPSPYQQNPTPSSGLGVRVGIKPEYRITPPPHLSSLAGDNPRSNFQFDFGLERKILAEAEKDNPNWSKFGSENIPTKVSDSSTAKVTALDPIVSKFMAMGLSREAVLIAVENYGDNPTKVHEFVNGYTLLREMGFSSNSVAEALAMNDNHTDKALAQLLNGSS

>G.max_04G239200.1
MDYDFRSRSGPQPPIYRPPPPPSPMYRPSPYQQNPTPSSGFGVRVGIKPEYRITPPPHLSSLAGDNPRSNFQFDFGLERKILAEAEKDNPNWSKFGSENVPTKVSDSSTAKVTALDPIVSKFMAMGLSQEAVPIAVENYGDNPTKVQEFVNGYTLLREMGFSSNSVADALVMNDNHTDKALAHFLNGSS

>P.vulgaris_008G159900.1
MDYDFRTGPPLYNSSSSPSSHQMYPKIAPHGHAAARPSHPHHSSPPSSSPGLGIRVFIKPEYRITPPPHLLPHSGDIQRSNFQFDFGLERKILSEGNKENPNWSKFGMENLPKVTESASPKVSVSDPIVSKYIAMGLSRDAVAIAVANYGDNPAKIPEFVKSYTLLREMGFSSTSVAEALVMYDNDTDKALAHFLNGSS

>P.vulgaris_009G136700.1
MVHAYSMDYDFRSRSGPQVPMYRPPPSPMYRPPPPPSSSPYQQNPTPSSGLGFGVRVAIKPEYRITPPPHLSSHAGDNSRSNFQFDFELERKILAEADKDNPNWSKFGSENIPTRVSDSSTAKVTALDSIVSKFIAMGLSQEAVPIAVENYGDNPTKVQEFVKGYTLLREMGFSSNSVAEALVLNDNRTDGALAHFLNGSS

>M.domestica_MDP0000144832
MEYDYRTRTGQMXTHRPATSSAPSSHPMYGPPSSSSSMYPRVGQQSHTAAPPPYGGNAGHPQHQPHHQTTNPPSSSSSGLGIRVTIKPEYRITPPPPLSLHVGDIPRSNFQFDFDLERKVLAELEKETPNWAKLGLENIPPPRAVEPPPSAGSGADPVVSKYIASGLSREAVPLAVANYGDNPTKVREFVNAFTLLREMGFSPNAVAEALMMYDNDTDKALAHFLNSSS

>M.domestica_MDP0000265089
MDYDYRSRSGQTPNYRPATSSAPSSHPMYGPPSSSSSMYPRVGQQSHAAVPPPYGGSAGRPLPHHQTANPPSSSSSGLGIRVTIKPDYRITPPPPLLLQVGDIPRSKFQFDFDLERKVLAELEKETPNWAKLGLQNLPPPRAVEPPSPSGSSADPVVSKYIASGLSREAVPLAVANHGDNPTKVREYVNAFTLLREMGFPQNAVAEALMMYDNDTDKALAHFLNNPS

> C.subellipsoidea_64252
MYPRINTGASGVLQVPSSGHANAYYPTVGVQSSPEFSQHEQGSAFSNGPAASGGSPTSQNFPLLRVQIAEQYRTLPPVMVSPGLDNIQQSSFTYNFDYERRIEAEDSAAASQESASGDDRSGSSNQAAASVQVEDPWTSQVLKYTEMGFSREEVCMALAALGTDADKDNEFMDFCKNYRELRSMGFPKATVAGALVAHTNDFAAATEACLAAQ

>M.pusilla_62799
MQQSNGAGVRLVIPKEYQLTPPVRAPRKKSPFNERGCERSIRCRVRRHPGNPTPDPPSSPPLPRTQVSLPRMDAEPPSSDAADFDTTRERNVLLEEGLEESPVAHENGGDDGRREPSEGRDPTGLEATVRRIVGAPDDPTVAEYVAGGHPRDAVTLGLAMWGSDKRDRVVQFCVGFEQMAGMGFRPEHVAGALASNDNDVEKAIAACLSESRSLPPGRH

> M.pusilla_59854
MNPDARPCVGVRIAVPATYQLTPPVSLPPLETSADAAADAADDARFDTTRERQVLAEETSPSTSPSPSPSTSARGAPADPTSLDATVRRVLDGPEDPTIEKFLRMGYPRDAVALGVAMWGDDPTKVPEFCTFFARGIEFGFPPHVLAGALAANDNNLEAAIGCCIK

**B) SEQUENCES USED FOR ALIGNMENT IN SUPPLEMENTAL DATASET 4**

>P.patens_PhPat.001G046300.1_NOG1

MEYDYGRSGHGSGGYEMGRPMYHSRQGSNVQGSYPRVGQSAGDALMNRGPPQAPLLSVPSFPSGSAIKVTIKPMYRLGPPAQLRVQSREVPRSLFQFEFDLERRILAEAEQGNLNFRAGSGATLSQSNSEADLAEVEDATVAKYLAMGHNKEAVQYALQTYGDDQNKVLDFCPPFNRIREMGFAADRVAKALASCNNDEEQAISSLVS

>M.polymorpha_0050s0076.1
MDFDYRKGGGGLTTSGRQQASGYDSNSRQPMYVPRYERSGSALVSQNQAYYPRIAQPVGPSVNRPPAPIPNNPSASNLPGSGIRVAIKPEYRVGPAVQLILPQNAEVPRSLFNFDFELERRILAEAERGAQGWDPARANGEQNLTPGMVDDPVVRKYLAMGLHREAVVMAVTTYGDVQNKVLDFVPSYNLLREMGFPAVAVTGALAMYDNDRERALAHFV

>S.fallax_0183s0013.1
MEQEYRKGGQTTRTQSSSGGGYDARPLYRMMTTSSQVNTDSSSSSFYPRIAQPVGGPPVNRPPPPPIAASNSFSSGTGIKVTIKPIYRVAPPVQLLPQGGEVPRSLFQFDFDLERRILAEAESQGQSFSRKPVDHSRQSNGGSVEDPTVAKYVSMGLNKEAVVMAIRTFGDIQNKVVDFCPTYSRLWEMGFQSDAVAAALATCDNDFERALAYLVSM

>S.fallax_0069s0038.1
MEYDYRKGGGGQSRSQSSSSGYDAARYNRLQSNNSSSSLYPRIAQPFGPSVNRPPPPPTIATSSSSSSGTGIKVTIKPIYQVAPPVQLLPQGGEVPRSLFQFDFDLERRILAAGESPGQPSYSTICADHSRLANGASASLAEVDDPAVGKYIAMGLNKEAVVMAIRTLGDVQNKVLDFCPSYDRLQEMGFQPDAVAAALAKSDIDFEQSLARLVSL

>S.moellendorfii_predicted

MEAAYDRRRYDQHHPTRPPPPNPSSSSFYPRLPQQAPAPPTVNRAALPPVPGAPPQSSGLGIKVALKPQYRMAPPVQLSPVIGEVPRTSIAQFDFDFERKVIDDAERGVRTQIRQHQLANGGETASASQANDDPVISKYLSMGLSREAVSLGVAAYGDVQAKVVEFCSAYSLLREMGFPSTAVAGALVMHDNDKEKALAQFV

>A.trichopoda_evm_27.model.AmTr_v1.0_scaffold00001.468
MDYDFRKGASPYDSSSAIPMYRPPSSYGAHGSPSYYPAPNRIAHTSGPPVSRPPPVPTTSSSPSSSLGIRVAIKPAYRITPPPQLSPQMAEIPRCSFLFDFEFERKILAEAEKESQNWSKIATETPSSSKPTESGTQESAADQLVNKYAALGLNREAVSFAVANYGDNANKVREFVSSYNLLREMGFASTNVARVLVMYDNDRDKALAHFLGSSV

>Z.marina_Zosma267g00330.1
MDYSYRHRNSYSPRAPPPPITSSMYPKMGSQPIQTDHAPPLPVRGSFAPQHNPPPPSSSTTGMGIRVSIKPEYRITSPPELFPQAKEVPQSKFHFDFEFERKFLAEIEKNGFCNWSKFSSEYQPLKDHTSSNSSMGAVNDVVVDKYTSSGLGREAVSFAVLNYGDNPVKVREFVKSYNLLREMGFVSKDVAEVLAMYDNDTDKALAHLLNTVKG

>S.polyrhiza_26G0010900
MDYDYRNRTGPVNRPAPTSSIYPRVSQQPGQHVPVPAGRATHPHPAAAPPTSGSGIIRVMLKPEYRITPPPQLAPQVTEVPRSHFNFDFEFERRILAEAEKDGQNWTRIVSENQSSFSASASSAGPAGDPVVEKYVAMGLGREAASLAVLSFGDDAIKVREFVKGYNLLREMGFASKNVAEALVMYDNDTDRALAHFLNSSS

>S.bicolor_Sobic.010G047400.1
MEYDFRGSRSGSGPGPYGAPPGAAPGGGSSLYPRVGQPSHGGGGASTASPRAAPYHHGSGSGSSAPVVTPLAPISSSSSSKVGIQVTIKPEFRIDPPPQLPPQMVEIPRSTFNFDFEYERRILAEAEKENPNWTKFVVERQAPPPVAQQARPASSGDPVVDRYVSMGLGREAVSFAVLNYGDNPTKVKEFVKSYNILHEMGFTSPNVPELLAIHDNDPDKVIQRLLSSPS

>S.viridis_4G063700.1
MEYDFRGRPGSGSYGAPPGAAAPGGGSSLYPRVGQPTHGGGGGGSATASPRAAPYHHGPGAGSGSSAPIVTPLAPTSTSSKVGIQVAIKPEFRITPPPQLPPQMVEIPRSTFNFDFEYERRILAEAEKENPNWSKFVVERQAPPPPVPQQARPASSGSGDPVVDKYVSMGLGREAVSFAVLNYGDNPAKVKEFVKSYNILHEMGFTSPNVPELLAIHDNDPDKVIQRLLSSPS

>B.distachyon_1g48630.2
MEYDFRGRPGSGSGSYAGSSGGGSSLYPRVGQPSHGGGSAPLQRPAPYLHPSAVASPAPNAPAPASSSSTSMGIQVVIKPEYRITPPPQLTPQMVEVSRSTFNFDFEYERKILAEAEKENPNWSKFVVERQTPPPPQPQPPRGPRHTTSTTSMGMQATPGDPVVQKYISMGLGREAVSFAVLNYGDNPTKVKEFVKSYNALHEMGFTSSNVPELLAMHDNDPDKVIQHLLSTP

>O.sativa_LOC_Os06g06530.1
MDYDYRGRPGSGSYGGGVGGGGGSSSLYPRVGQPSHGVANAPPPEPPRAAPYHHHGPPTVSAAPHPVPASSSTSMGIQVVIKPAYRITPPPQLPPQLTEIPRSTFNFDFEYERKILAEAEKENPNWSKFVIESQPPPPPQPPRGPKLTTPTTSVATPGDPVVDKYISMGLGREAVSFAVLNYGDNPAKVKEFVKSYNALHEMGFTSSNVPELLAIHDNDPDKVIQHLIGTS

>Z.mays_GRMZM2G071249_T01
MEYDFRGGRSSSGPGPYGAPPGGAPGGGSSLYPRVGQPSHGGGGASTASPRAAPYHHGSGSGSGSGSSAPVVTPLAPTSFSSSSSSSSKVGIQVAIKPEFRITPPPQLPPEMVEIPRSTFNFDFEYERMILAEAEKENPNWTKFVVERQAAPLVPQQARPASSGSGDPVVDKYVSMGLGRQAVSFAVLNYGDNPTKVKEFVKSYSILHEMGFTSPNVPELLAIHDNDPDKVIQRLLSSPS

>P.hallii_J00097.1
MEYDYRGRPGSGSYGAAPGGGGGGPSLYPHVGQPSHGGGGSGTASPRAAPYHHGPGGGSGSSAPIVTPLAPTSTSSSKVGIHVAIKPEFRITPPPQLPPQMVEIPRSTFNFDFDYERRILAEAEKENPNWSKFVVERQAPPPVPQQQARPASSGSGDPVVDKYVAMGLGREAVSFAVLNYGDNPAKVKEFVKSYNILHEMGFTSPNVPELLAIHDNDPDKVIQRLLSSPS

>S.lycopersicon_03g007520.2.1
MEYDFRNRTGPPYDTQSPMYGRPATGAPQPHPMYGQAPGLYPRPGQHSGGRNPPFHHTPPPSSNTGIGIRVAIKPEYRITPPPQLSTQVGEIPRSTFNFDFDLEKKILAEAEKESQNWSKLGLENLPPIMPEQPSMGYTGDLMMNKYATAGFNREAVAIAVANYGDNPIKVKEFVEGYTLLKEMGFSSNSVADALLMNDNDKDKAAAQLLGNSS

>S.lycopersicon_06g036500.1.1
MEPSYSTQSPMYSRPATTALPLTIHHSPMYGQASAEIGIRIAIIPEFQITPPPPLVPQGDVPQCTFQFDFDLEKKILAEANQKESQIWSRLPIENLPSRGSDQASKVHFHVNL

>S.tuberosum_PGSC0003DMT400023416
MEYDFRNRTGPPYDTQLPMYGRPATGAPHPHPNPQPHPMYGQAPGLYPRPGQHSGVRNPPFHHIPPPSSNTGIGIRVAIKPEYRITPPPQLSTQVGEIPRSTFNFDFDLEKKILAEAEKESQNWSRLGLENLPPRMPEQTNMGYTGDPMMNKYATSGFNREAVAIAVANYGDNPIKVKEFVEGYTLLKEMGFSSNSVADALMMNDNDKDKAAAQLLGNSS

>S.tuberosum_PGSC0003DMT400074355
MAYDLRNRTDPSYGTQSPKYGRPATTAPPLPSHHHPMYGQPSSLYPRVGQHSGGNFPGRFNPTPSPSSTTGIGIRVAIKPEYQITPPPPLAPQGDVPQCTFQFDFDLEKKILAEADQKESQIWSRLPLENLPSRGSDSDQASKASSGDPVMIKYIAFGLNREAVPLAVANYGDNPNKVTEFADGYTYLKEMGFSSNSAADALLMNDNDTNKAIQHLLNNPSY

>P.trichocarpa_015G023600.1
MDYDFRNRTSSPYDTPSTMHRSSTPSTAPQPSHPMYGPPSLYPTVNQPGHTVIPHAPRHHSFTQQAPSSPSSGLGIRVMIKPEYRITPPPQLTPQIVEIPRSSFQFDFELERQILAEAEKDSPNWSRLLGLENSPPKPPPILGSSLIVGKYPVVRKYISMGLNRDAVPLAVANYGDNPPKVQEFVNGYTLLQEMGFPSNKVAEALLMYDNNTDEALAHFLNSS

>P.trichocarpa_012G033300.1
MEYDFRNRTSSPYDTQSPMYRSSTPLTTAPPPTHPMYGPSLYPRVSQPAHPAIPPVSRHHSFPQPSSSSPSSGLGIRVMIKPEYRITPPPQLTPQIGEIPRSSVQFDFELERQIIAEAEKGSVNWSRLLGLENLPSKPLESTPSTGPTADPVERKYIASGLSRDAVPLAVANYGDNPTKVQEFVNGYTLLREMGFSSSSVAEALLTYDNDADKALAHFLGSS

>G.raimondii_007G014400.1
MDYDFRNRTGPPYEAQIPIYRQQPTSSSSTHPMYGSSMYPRIGGQPAAHSVAPPTTRASSFHQNFSPSSSSGLGIRVALKPKYRITPPPQLSPQVGDIPRSNFQFDFEFERKILAEAEKENMNWSKLGLENLSSKPIETSSSSTGANSDPVVSKYIASGLSREAVTLAVANYGDNPTKVREFVTGYNLLREMGFSSNNVAEALLMYDNDTDKALAHFLNSSS

> G.raimondii_006G200100.1
MYRQQLTSTSSSSSRPMYRPSIYPNVGQPGHPVVPPAPRTSSSPSSSAGLGIRVVLKPDYRITPLPQFSPQVGDIRRSNFQFDFEFERKILAQPDAEFMNLSQLDLENHPSEPTQSTPSSGANSDSVLNKYIASGLSQEAVIIAVANYGDSPTKVREFVNGYNLLREMGFSANNVADALLVCDNDTDKALAHFLNSSS

>T.cacao_1EG015327t1
MDYDFRNRTGPPYEAQIPMYRQQPTSSSSSSSMPSSHPMYGPSLYPRIGQPAHTVVPPAPRIPSFHHTSSPSSSSGLGVRVALKPEYRITPPPQLLPQVGDIPRSNFQFDFGFERKILAEAENESMNFSRLGLESLSKSTESTSSSGANADPVVSKYIASGLNREAVTVAVANYGDNPTKVREFVHSYNLLREMGFSSNNVAEALLMYENDTDKALAHFLNNSS

>A.thaliana_AT5G53330.1
MDYDYRNKSGGPSYPRPMYGPPSTSPSPSSNHPMYGYPKIGQQTGPGPQFFSPPERNSSFQHNTSPSSGIGIRVNLKPEYRITPPPQLLPRVGDIHRSSFQFDFGLERKVLAEAEKDNPDWSKFGSENPPAKFHEPSPSSVGQMQGVDHVVMKYTASGLNREAVNIAVANYGDNPTKVQEFANGFTAIREMGFPTNAVADALFMFENDTDKALAHLLHGSS

>B.rapa_J00835.1
MDYDYRNKSGPSYARPMYGYPNIGQQSGHGHQFFPPPERNQSFQQHNSSPFPFSSSSSSSGLGIKVTLKPEFRITPPPQLLPRAGDIPRSGFQFDFGLERAVLTEAEKDNPDWSKFGSDIPPPSNFPQPPPVPSMGVDPLVMKYTASGLNREAVNIAVANYGDNPTKVQEFANGFAAMREMGFPTNAVAEALFMFDNDTDKALSHLLHGSS

>B.rapa_C01396.1
MDYDFRNKSGPPYGRPMYPSPSPSSTHPMYNGPPGYPKIGQQSSHGQPFFPPPERNPSFQHNPSPSSSSGLGIKVTLKPEYRITPPPPLLPRVGDVPRSSFQFDFGLERKILAEAEKENPDWSKFGSEHPPPPSNFPPTPSPSMGVDPVVMKYAGLNREAVNIAVANYGDNPTKVQEFANGFTALREMGFPTNAVADALFMFENDTEKALSHLLHGSS

>B.oleracea_030304
MDYDYRNKFGASYARPMYGPPSISPSPPSTHPMYGYPNIGQQSGHGHQFFPPPERNQSFQHNSSPSPFSSSSGLGIKVTLKPEYRITPPPQLLPRAGDIPRSGFQFDFCLERAVLAEAEKDNPDWSKFGSDIPPPSNFPQPPPVPSMGVDPLVMKYTASGLNREAVNIAVANYGDNPTKVQEFANGFAAMREMGFPTNAVAEALFMFDNDTDKALSHLLHGSS

>B.oleracea_027997
MDYDFRNKSGPPYGRPMYPSPSPSSTHPMYNGPPGYPKIGQQSSHGQPFFPPPERNPSFQHNPSPSSSSGLGIKVALKPEYRITPPPPLLPRAGDIPRSSFQFDFGLERKVLAEAEKENPDWSKFGSEHPPPPSSFPPTPSPSMGVDPVVMKYAGLNREAVNIAVANYGDNPTKVQEFANGFTALREMGFPTNAVSDALFMFENDTEKALSHLLHGSS

>C.rubella_10027076m
MDYDYRNKSGPSYPRPMYEPSSASSPSPASNHPMYAPSGYPKIGQQTGHGQQFFPPPERNSSFQHNTPPPFPSSSSSSGLGIKVNLKPEYRITPPPQLLPRVGDIHRSSFQFDFGLERKVLAEAEKDTPDWSKFGTENPPAKFPEPSSSSMGVDPVMKYAGLNREAVNIAVANYGDNPTKVQEFANGFTAIREMGFPPNSVAEALFMFENDTEKALAHLLHGSS

>E.salsugineum_10014603m
MDYDFRNKSGPPYARPMYGTASASPSPPSSHSMYGPPGYPKIGQQSGHGQLFFPPPERNSSFQHNSSPFPSSSSSGLGIKVTMKPEYRITPPPQLLPRVADIPRSSFQFDFSLEKKVLAEAEKENPDWSKFGTENPPQAKFPEPAPSMGVDPIVMKYAGSGLNREAVNIAVANYGDNPTKVQEFANGFSAIREMGFPTNAVADALFMFENDTEKALAHLLHGSS

>M.trunculata_3g090640.1
MEYEYNRSRSGPQVPMYRAPPSIYPKIGPHPHSAAPRPPPFQHQNPNPSPSIGLGIKVAIKPEYKIAPPPHLLPHVGDIPRSNFQFDFGLERKILAEAEKENPNWTKFGVENLPTKASDSSPSSKVTTADPIVNKYIAMGLSRDVVPIAVKNYGDNPTKVQEFVKGYTLLHEMGFSSNSVDEALLMYDNDTDKALAYFLNGSS

>G.max_06G124600.1
MDYDFRSRSGPQPPMYRPPPPPSPMYRPSPYQQNPTPSSGLGVRVGIKPEYRITPPPHLSSLAGDNPRSNFQFDFGLERKILAEAEKDNPNWSKFGSENIPTKVSDSSTAKVTALDPIVSKFMAMGLSREAVLIAVENYGDNPTKVHEFVNGYTLLREMGFSSNSVAEALAMNDNHTDKALAQLLNGSS

>G.max_04G239200.1
MDYDFRSRSGPQPPIYRPPPPPSPMYRPSPYQQNPTPSSGFGVRVGIKPEYRITPPPHLSSLAGDNPRSNFQFDFGLERKILAEAEKDNPNWSKFGSENVPTKVSDSSTAKVTALDPIVSKFMAMGLSQEAVPIAVENYGDNPTKVQEFVNGYTLLREMGFSSNSVADALVMNDNHTDKALAHFLNGSS

>P.vulgaris_008G159900.1
MDYDFRTGPPLYNSSSSPSSHQMYPKIAPHGHAAARPSHPHHSSPPSSSPGLGIRVFIKPEYRITPPPHLLPHSGDIQRSNFQFDFGLERKILSEGNKENPNWSKFGMENLPKVTESASPKVSVSDPIVSKYIAMGLSRDAVAIAVANYGDNPAKIPEFVKSYTLLREMGFSSTSVAEALVMYDNDTDKALAHFLNGSS

>P.vulgaris_009G136700.1
MVHAYSMDYDFRSRSGPQVPMYRPPPSPMYRPPPPPSSSPYQQNPTPSSGLGFGVRVAIKPEYRITPPPHLSSHAGDNSRSNFQFDFELERKILAEADKDNPNWSKFGSENIPTRVSDSSTAKVTALDSIVSKFIAMGLSQEAVPIAVENYGDNPTKVQEFVKGYTLLREMGFSSNSVAEALVLNDNRTDGALAHFLNGSS

> C.subellipsoidea_64252
MYPRINTGASGVLQVPSSGHANAYYPTVGVQSSPEFSQHEQGSAFSNGPAASGGSPTSQNFPLLRVQIAEQYRTLPPVMVSPGLDNIQQSSFTYNFDYERRIEAEDSAAASQESASGDDRSGSSNQAAASVQVEDPWTSQVLKYTEMGFSREEVCMALAALGTDADKDNEFMDFCKNYRELRSMGFPKATVAGALVAHTNDFAAATEACLAAQ

>M.pusilla_62799
MQQSNGAGVRLVIPKEYQLTPPVRAPRKKSPFNERGCERSIRCRVRRHPGNPTPDPPSSPPLPRTQVSLPRMDAEPPSSDAADFDTTRERNVLLEEGLEESPVAHENGGDDGRREPSEGRDPTGLEATVRRIVGAPDDPTVAEYVAGGHPRDAVTLGLAMWGSDKRDRVVQFCVGFEQMAGMGFRPEHVAGALASNDNDVEKAIAACLSESRSLPPGRH

> M.pusilla_59854
MNPDARPCVGVRIAVPATYQLTPPVSLPPLETSADAAADAADDARFDTTRERQVLAEETSPSTSPSPSPSTSARGAPADPTSLDATVRRVLDGPEDPTIEKFLRMGYPRDAVALGVAMWGDDPTKVPEFCTFFARGIEFGFPPHVLAGALAANDNNLEAAIGCCIK
