# Supplementary material for: Genetic Regulation of the 2D to 3D Growth Transition in the Moss Physcomitrella patens
Source: Curr Biol. 2018 Feb 5;28(3):473–478.e5. doi: 10.1016/j.cub.2017.12.052 (PMC5807088; doi:10.1016/j.cub.2017.12.052)
Supplement: Document S2. Article plus Supplemental Information [file mmc6.pdf]

Report

Current Biology

Genetic Regulation of the 2D to 3D Growth Transition in the Moss *Physcomitrella patens*

Highlights

- *NO GAMETOPHORES 1* (*PpNOG1*) regulates the 2D to 3D growth transition in *P. patens*
- PpNOG1 acts upstream of 3D-promoting PpAPB transcription factors
- PpNOG1 is required for the formation of apical initial cells specified for 3D growth
- *NOG1* genes are found only in land plants and thus evolved coincident with 3D growth

Authors

Laura A. Moody, Steven Kelly, Ester Rabbinowitsch, Jane A. Langdale

Correspondence

jane.langdale@plants.ox.ac.uk

In Brief

Moody et al. use forward genetics, somatic hybridization, and genome sequencing to identify *NO GAMETOPHORES 1* (*PpNOG1*), a gene that regulates the two-dimensional (2D) to three-dimensional (3D) growth transition in the moss *Physcomitrella patens.* PpNOG1 promotes the formation of apical initials that are required for the establishment of 3D growth.

Moody et al., 2018, Current Biology *28*, 473–478
February 5, 2018 © 2018 The Authors. Published by Elsevier Ltd.
https://doi.org/10.1016/j.cub.2017.12.052

Current Biology
Report

Genetic Regulation of the 2D to 3D Growth Transition in the Moss *Physcomitrella patens*

Laura A. Moody,[1] Steven Kelly,[1] Ester Rabbinowitsch,[1] and Jane A. Langdale[1,2,*]
[1]Department of Plant Sciences, University of Oxford, South Parks Rd., Oxford OX1 3RB, UK
[2]Lead Contact
*Correspondence: jane.langdale@plants.ox.ac.uk
https://doi.org/10.1016/j.cub.2017.12.052

SUMMARY

One of the most important events in the history of life on earth was the colonization of land by plants; this transition coincided with and was most likely enabled by the evolution of 3-dimensional (3D) growth. Today, the diverse morphologies exhibited across the terrestrial biosphere arise from the differential regulation of 3D growth processes during development. In many plants, 3D growth is initiated during the first few divisions of the zygote, and therefore, the genetic basis cannot be dissected because mutants do not survive. However, in mosses, which are representatives of the earliest land plants, 3D shoot growth is preceded by a 2D filamentous phase that can be maintained indefinitely. Here, we used the moss *Physcomitrella patens* to identify genetic regulators of the 2D to 3D transition. Mutant screens yielded individuals that could only grow in 2D, and through an innovative strategy that combined somatic hybridization with bulk segregant analysis and genome sequencing, the causative mutation was identified in one of them. The *NO GAMETOPHORES 1* (*NOG1*) gene, which encodes a ubiquitin-associated protein, is present only in land plant genomes. In mutants that lack PpNOG1 function, transcripts encoding 3D-promoting *PpAPB* transcription factors [1] are significantly reduced, and apical initial cells specified for 3D growth are not formed. *PpNOG1* acts at the earliest identified stage of the 2D to 3D transition, possibly through degradation of proteins that suppress 3D growth. The acquisition of NOG1 function in land plants could thus have enabled the evolution and development of 3D morphology.

RESULTS AND DISCUSSION

To discover novel regulators of 3D growth, we designed a forward genetic screen to identify mutants of the moss *Physcomitrella patens* that could grow as 2D filaments, but not as 3D shoots. In wild-type (WT) *P. patens*, haploid spores germinate to produce apical initials that divide in a single plane to generate filaments of chloronemal and then caulonemal cells that are collectively referred to as protonema [2–4] (Figure 1A). Caulonemal cells divide either in a single plane to extend the filaments or in two planes to form side-branch initials. Most side-branch initials develop into secondary caulonemal filaments (Figure 1A), but ~5% are specified to cleave in three planes, producing 3D leafy shoots known as gametophores (Figure 1B). In a primary screen of 9,000 UV-mutagenized lines of the Villersexel (Vx) strain [5] of *P. patens*, 26 mutants were identified that developed normal 2D filaments but were unable to form 3D gametophores. A secondary screen discarded 12 of those mutants because plants entirely comprised chloronemal filaments and were therefore likely to be defective in the chloronemal-to-caulonemal rather than the 2D to 3D growth transition. Because gametophores are induced by cytokinin and auxin in wild-type *P. patens* (Figure S1), and mutants defective in the respective signaling pathways fail to form gametophores [6–8], a tertiary screen was carried out to further eliminate mutants in the cytokinin biosynthesis pathway. Another 12 mutants that formed normal gametophores when exposed to the cytokinin analog 6-benzylaminopurine (BAP) were discarded at this stage. One of the remaining mutants, *P. patens no gametophores 1 - Reference* (*Ppnog1-R*), exhibited normal 2D growth, with no detectable defects in protonemal tip growth or in branching (Figures 1C and 1D), but the formation of gametophores was completely abolished, even in the presence of cytokinin and/or auxin (Figures 1E and S1). *Ppnog1-R* mutants are thus unable to establish 3D growth.

Because the *Ppnog1-R* mutant does not produce gametophores, egg-producing archegonia and sperm-producing antheridia cannot develop, and thus the causative mutation could not be mapped by conventional genetic crosses. A novel strategy was therefore designed for gene discovery. As a first step, a previously published technique [9] was used to generate somatic hybrids between the infertile *Ppnog1-R* mutant and fertile lines of the Gransden (Gd) strain of *P. patens*. The presumed diploid hybrids produced phenotypically normal gametophores that generated sporophytes after fertilization (Figures 2A–2C). Spores obtained from three independent *Ppnog1-R*/Gd hybrid sporophytes exhibited phenotypic segregation ratios consistent with meiosis from a tetraploid, confirming that the original hybrids (and the generated spores) were diploid (Figures 2A, 2B, and 2D). Genomic DNA was extracted from diploid segregants (120 mutant, no 3D growth; 120 wild-type, 3D growth) and sequenced in two separate pools alongside both Vx and Gd parental lines. When allele frequencies for the 120 mutant individuals were plotted across all 27 chromosomes in the *P. patens* genome assembly, a single allele

This is an open access article under the CC BY license (http://creativecommons.org/licenses/by/4.0/).

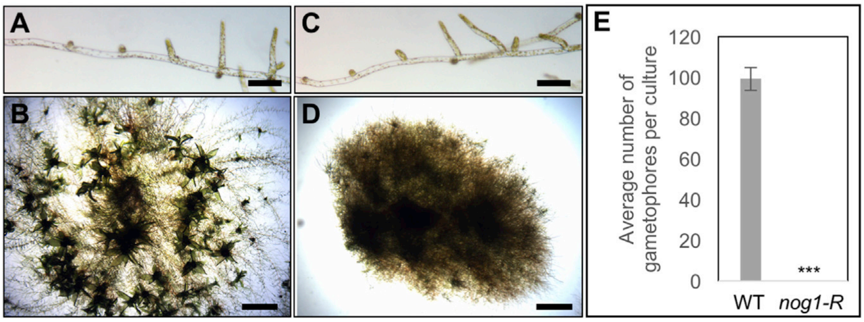

Figure 1. 3D Growth Is Abolished in *Ppnog1-R* Mutants

(A–D) 7-day-old (A and C) and 1-month-old (B and D) wild-type (WT) (A and B) and *Ppnog1-R* (C and D) plants showing protonemal filaments (A and C) and the presence (WT, B) or absence (*Ppnog1-R*, D) of gametophores.

(E) Mean number of gametophores/culture (n = 10) ± SEM (WT = 99.4 ± 5.62; *Ppnog1-R* = 0 ± 0; t test $p < 0.05$ ***).

Scale bars, 100 μm (A and C) and 1 mm (B and D).

See Figure S1 for response to cytokinin and auxin.

frequency peak of 1 was revealed on chromosome 1, identifying the genetic interval containing the *Ppnog1-R* locus (Figures 2C and 2E). The allele frequency plot for the 120 wild-type individuals revealed a mutant allele frequency of 0.4 at the corresponding location, representing the expected frequency from a mixture of wild-type homozygous and heterozygous individuals within the pool (Figures 2D and 2F). Interrogation for SNPs and other deviations from the annotated genome sequence (*P. patens* V3.3) in the genetic interval that contained the *Ppnog1-R* locus, revealed mutations in the coding or regulatory regions of four annotated genes (Figure 2E). Of the four mutations identified, we postulated that the *Ppnog1-R* mutant phenotype was caused by a C > T transition that generated a premature termination codon (R^{76}Ter) in a gene (#32970008) that encodes a ubiquitin-associated protein (Figure 2E).

To validate that mutations in gene #32970008 caused the *Ppnog1-R* phenotype, we carried out complementation experiments. Three splice variants of the transcript were detected in the *Ppnog1-R* mutant, and all resulted in the introduction of a premature termination codon into the coding sequence (Figures 3A and 3B). The mutant phenotype was complemented when a full-length coding sequence was introduced into the genome (Figures 3C–3F and S3A; Data S1A), and thus the gene was named *PpNOG1*. Given the likely presence of

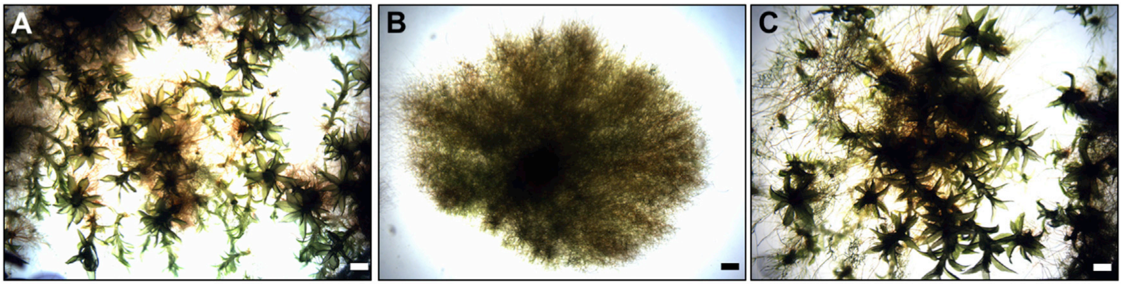

D

	Observed			Expected				
	3D growth	No 3D growth	Total	3D growth	No 3D growth	Total	Chi-square	Percentage
Ppnog1/Gd-2	500	108	608	506.67	101.33	608	0.526 ***	17.7631579
Ppnog1/Gd-3	468	103	571	475.83	95.17	571	0.773 ***	18.0385289
Ppnog1/Gd-8	564	99	663	552.5	110.5	663	1.436 ***	14.9321267

E

Chromosome	Position	Reference	Variant	Gene	Feature	Amino Acid Change
Chr01	8286060	C	T	32970008	exon_2	CGA (Arg) to UGA (stop)
Chr01	8415703	GTG	GAGGTG	32968676	exon_2	+ GAG (Glu)
Chr01	8836609	TTTTC	T	32967989	five_prime_UTR	N/A
Chr01	7511627	TTATATATATATA	T	32967881	three_prime_UTR	N/A

Figure 2. Identification of the *PpNOG1* Locus through Combined Somatic Hybridization and Bulk Segregant Analysis

(A–C) Representative phenotype of 1-month-old wild-type Vx plant with gametophores (A), *Ppnog1-R* plant lacking gametophores (B), and *Ppnog1-R*/Gd hybrid exhibiting restored gametophore formation (C). Scale bars, 1 mm.

(D) Phenotype of progeny derived from three independent *Ppnog1-R*/Gd hybrid sporophytes. Observed numbers are consistent with the hybrid gametophores being diploid, and the fertilized sporophytes being tetraploid. Chi-square test; $p < 0.05$ ***.

(E) Candidate genes in the genetic interval containing the *PpNOG1* genetic locus. The C > T transition in gene 32970008 (red) generated a premature stop codon. See Figure S2 for overview of strategy.

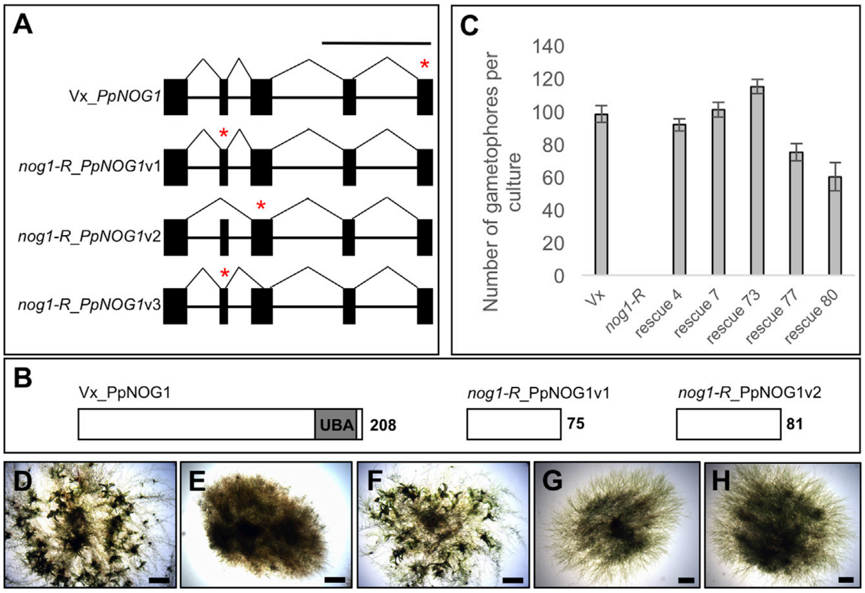

Figure 3. A Premature Stop Codon in *PpNOG1* Abolishes 3D Growth

(A) *PpNOG1* transcripts in parental Vx and *Ppnog1-R*. Blocks, exons; asterisks, in-frame stop codon. Scale bar, 1 kb.

(B) PpNOG1 protein in Vx contains a ubiquitin-associated domain (UBA), which is missing in *Ppnog1-R*. # amino acid residues indicated.

(C) Mean number of gametophores/culture (n = 10) in Vx, *nog1-R*, and five independent *Ppnog1-R* lines complemented with full-length *PpNOG1* cDNA. Error bars ± SEM.

(D–G) 1-month-old Vx (D), *Ppnog1-R* (E), complemented *Ppnog1-R* (F), *Ppnog1-D1* (G), and *Ppnog1-D2* (H) disruptant mutants. Scale bars, 1 mm.

See Figure S3 and Data S1 for details of complementation and disruptant constructs.

additional UV-induced mutations in the *Ppnog1-R* mutant, further validation was obtained by generating disruptant lines in which the *PpNOG1* genomic locus in the Vx strain was disrupted with a *PpNOG1* transcript containing a premature termination codon (R^{76}Ter; Figures 3B and S3B–S3E; Data S1B). Two disruptant mutants were obtained (*Ppnog1-D1* and *Ppnog1-D2*), in which there was no transition from 2D to 3D growth (Figures 3G and 3H). Intriguingly, the recombination event in the *Ppnog1-D1* line was such that both the mutant and native *PpNOG1* sequences could be transcribed (Figures 3B–3E), suggesting that the truncated protein had a dominant-negative effect. However, this effect is not seen in the diploid *Ppnog1-R*/Gd hybrid (Figure 2C) or in the complemented *Ppnog1-R* lines (Figure 3F), where gametophores form normally. As such, the dominant negative effect most likely reflects the relative levels of mutant versus native transcript in each case. In disruptant, diploid, and rescue lines, the mutant transcript is driven by the endogenous *PpNOG1* promoter, but the native transcript is driven by either a relatively short *PpNOG1* promoter sequence introduced by the transformation construct (disruptant) (Figure S3E), the endogenous *PpNOG1* promoter (diploid) or the actin promoter (rescue). Therefore, when the native transcript can accumulate to the same level as the mutant transcript, the mutant allele behaves as a recessive. Together, these results confirmed that a premature termination codon in the *PpNOG1* gene prevents 3D growth.

Although gametophore development is completely abolished in *Ppnog1-R, D1* and *D2* mutants, bulbous side branch cells characteristic of gametophore initiation were occasionally observed (at ~10% frequency of wild-type) (Figures 4A and 4E). Unlike in wild-type (Figures 4B and 4C), however, the first division plane in these "bud" initials was not oblique (Figure 4F) and cell plates were positioned randomly during subsequent divisions (Figures 4G–4L). Similar misplacement of cell division planes is seen when the *TONNEAU* gene is mutated in *P. patens*, but in *Ppton1* mutants (which cannot form preprophase bands), recognizable but distorted 3D leafy shoots are observed [10]. In contrast, a tetrahedral gametophore initial that can cleave in three planes and establish 3D growth is never formed in *Ppnog1* mutants, and with few exceptions development arrests at the five-cell stage. Although the arrested phenotype is similar to that seen when the calpain protease *PpDEK1* is mutated [11, 12], there are important distinctions between the two mutant phenotypes. The first is that supernumerary bud initials form on *Ppdek1* mutant caulonema [11], indicative of opposing roles during the initiation of 3D growth, with *PpDEK1* acting to repress the 2D to 3D transition and *PpNOG1* acting to promote it. The second is that the oblique cell division that distinguishes 3D-forming bud initials from 2D branch initials is positioned correctly in *Ppdek1* mutants [11], suggesting that PpNOG1 acts earlier than PpDEK1 during 3D specification. Together, the substantially reduced number of bud initials, the misplaced oblique cell division plane in the bud initials that do form, and the complete absence of gametophores in *Ppnog1* mutants suggest that PpNOG1 plays a critical role in promoting the transition from 2D to 3D growth in *P. patens.*

Four *PpAPB* genes that encode AP2-like transcription factors are both necessary and sufficient in *P. patens* for the formation of bud initials that can develop into gametophores [1], and as such it is important to understand the relationship between PpAPB and PpNOG1 function. *PpAPB* [1] and *PpNOG1* (Figure 4M) transcript levels are both induced by auxin, with *PpAPB* genes showing the more dramatic response. In contrast, *PpNOG1* transcript levels show no consistent response to cytokinin (Figure 4M), suggesting a role in specification of 3D growth per se as opposed to a role in the cytokinin response pathway. Notably, the accumulation of *PpAPB* transcripts was significantly reduced in both *Ppnog1-D1* and *Ppnog1-D2* disruptants (Figures 4N and 4O). Collectively these results suggest that the genes act in the same pathway and that at the level of transcription, *PpNOG1* acts upstream of *PpAPB* genes. However, interactions are unlikely to be direct. The presence of a ubiquitin associating domain in PpNOG1 may indicate a role in protein degradation, particularly given that at least one ortholog in flowering plants (Figure S4) is known to bind ubiquitin [13]. With *PpDEK1*

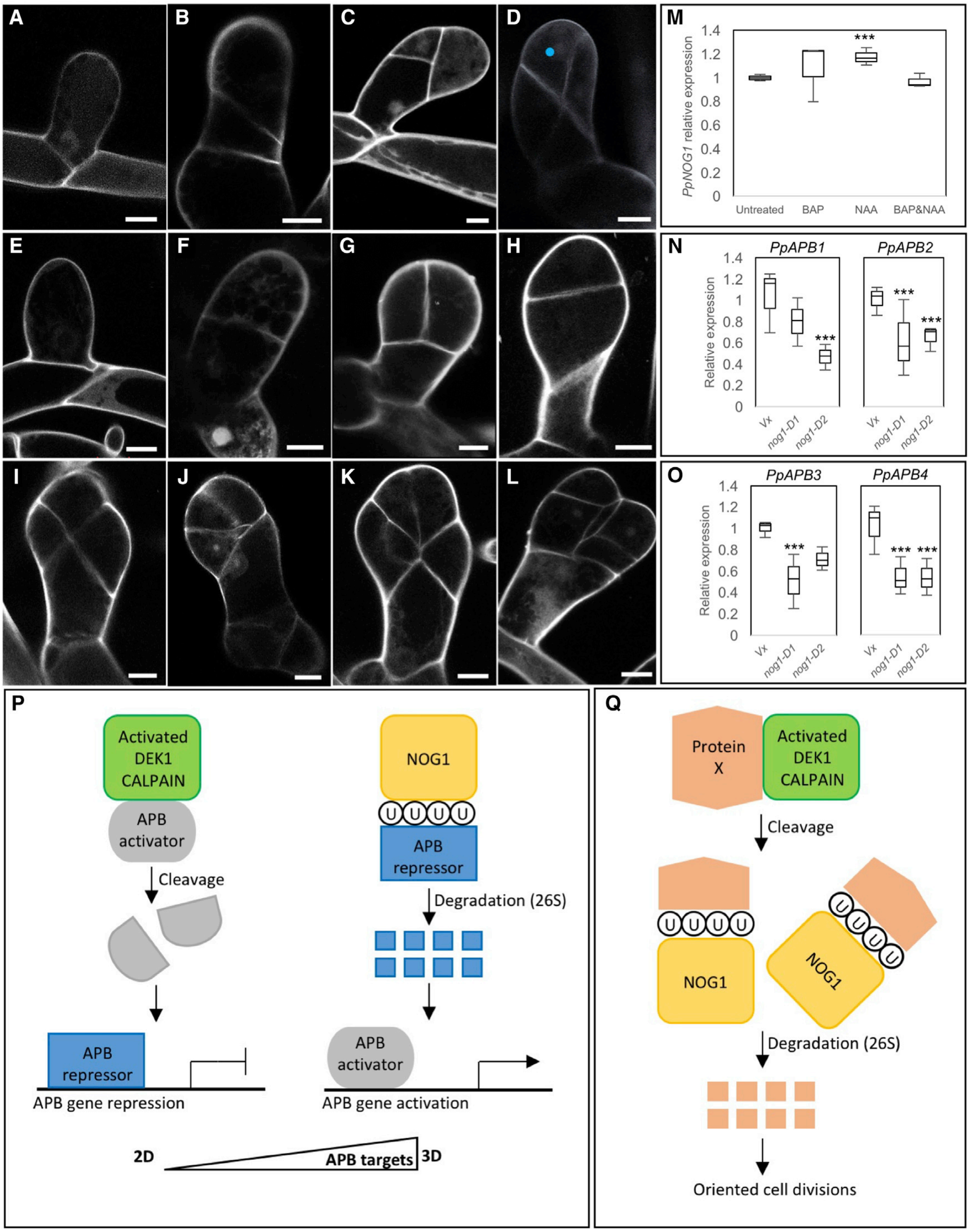

Figure 4. PpNOG1 Function

(A–L) Propidium iodide stained Vx (A–D) and *Ppnog1-R* (E–L) side-branch cells (A and E) and buds at 2- (B and F), 3- (C, G, and H), 4- (D, I, and J), and 5- (K and L) cell stages. Scale bars, 10 μm. ●, gametophore initial.

(M–O) Relative transcript levels in protonemata: *PpNOG1* in wild-type ± BAP and/or NAA (M) and *PpAPB1-4* in Vx and the *Ppnog1-D1* and the *Ppnog1-D2* disruptants (N and O). ANOVA, ***$p < 0.05$.

(P and Q) Model for PpNOG1 function: PpDEK1 and PpNOG1 act antagonistically during side-branch initiation to regulate *PpAPB* transcription (P) and then act together to enable divisions that produce the gametophore initial (Q).

See Figure S4 for NOG1 phylogeny.

encoding a membrane-bound protease [14], and mutants in both proteasome [15] and N-end rule [16] degradation pathways perturbing (but not abolishing) gametophore development, post-translational regulation is emerging as a common theme in the transition from 2D to 3D growth. In this context, we propose that PpNOG1 regulates the transition to 3D growth by first inducing the degradation of protein(s) that repress PpAPB activity (and hence opposing PpDEK1 activity by inducing caulonema to produce bud initials) (Figure 4P), and then by acting within the bud initial (alongside PpDEK1) to ensure that cell divisions are correctly orientated to produce an apical initial that can cleave in three planes and self-renew (Figure 4Q). Given that *NOG1* sequences are absent from all non-plant genomes (Figure S4), it is plausible that this role evolved to facilitate 3D growth in plants and thus that the antagonistic relationship between NOG1 and DEK1 likely represents the earliest regulatory module associated with 3D growth in both developmental and evolutionary terms.

STAR★METHODS

Detailed methods are provided in the online version of this paper and include the following:

- KEY RESOURCES TABLE
- CONTACT FOR REAGENT AND RESOURCE SHARING
- EXPERIMENTAL MODEL AND SUBJECT DETAILS
  - Plants
- METHOD DETAILS
  - UV mutagenesis and screening
  - Microscopy
  - Somatic hybridization and segregation analysis
  - Isolation of genomic DNA and sequencing
  - Read processing and variant calling
  - Candidate gene discovery through bulk segregant analysis
  - Physcomitrella patens transformation
  - Generation of nog1-R complementation lines
  - Generation of a nog1 disruptant mutant
  - DNA gel blot analysis
  - Quantitative RT-PCR
  - Phylogenetic analysis
- QUANTIFICATION AND STATISTICAL ANALYSES
  - Experimental design, sampling and statistical methods
- DATA AND SOFTWARE AVAILABILITY

SUPPLEMENTAL INFORMATION

Supplemental Information includes four figures and four data files and can be found with this article online at https://doi.org/10.1016/j.cub.2017.12.052.

ACKNOWLEDGMENTS

We are grateful to Pierre-François Perroud for providing the Vx::mCherry and the Gd::GFP marker lines; Yuji Hiwatashi, Rumiko Kofuji, and Mitsuyasu Hasebe for providing pZAG1 and pGFPmutNPTII plasmids; Andrew Cuming for advice on UV-mediated mutagenesis; and Jill Harrison for helpful comments on the manuscript. The work was funded by ERC Advanced Investigator (EDIP) and BBSRC (BB/M020517/1) grants (to J.A.L.) and by a Royal Society University (UF140484) Research Fellowship (to S.K.).

AUTHOR CONTRIBUTIONS

L.A.M. performed all of the experimental work, with technical assistance from E.R.; L.A.M., S.K., and J.A.L. conceived and designed the study; S.K. carried out the bioinformatics; and L.A.M. and J.A.L. wrote the manuscript, with editorial input from S.K.

DECLARATION OF INTERESTS

The authors declare no competing interests.

Received: November 18, 2017
Revised: December 20, 2017
Accepted: December 22, 2017
Published: January 25, 2018

REFERENCES

1. Aoyama, T., Hiwatashi, Y., Shigyo, M., Kofuji, R., Kubo, M., Ito, M., and Hasebe, M. (2012). AP2-type transcription factors determine stem cell identity in the moss *Physcomitrella patens*. Development *139*, 3120–3129.
2. Cove, D., Bezanilla, M., Harries, P., and Quatrano, R. (2006). Mosses as model systems for the study of metabolism and development. Annu. Rev. Plant Biol. *57*, 497–520.
3. Harrison, C.J., Roeder, A.H., Meyerowitz, E.M., and Langdale, J.A. (2009). Local cues and asymmetric cell divisions underpin body plan transitions in the moss *Physcomitrella patens*. Curr. Biol. *19*, 461–471.
4. Kofuji, R., and Hasebe, M. (2014). Eight types of stem cells in the life cycle of the moss *Physcomitrella patens*. Curr. Opin. Plant Biol. *17*, 13–21.
5. Perroud, P.-F., Cove, D.J., Quatrano, R.S., and McDaniel, S.F. (2011). An experimental method to facilitate the identification of hybrid sporophytes in the moss *Physcomitrella patens* using fluorescent tagged lines. New Phytol. *191*, 301–306.
6. Ashton, N.W., Cove, D.J., and Featherstone, D.R. (1979). The isolation and physiological analysis of mutants of the moss, *Physcomitrella patens*, which over-produce gametophores. Planta *144*, 437–442.
7. Ashton, N.W., Grimsley, N.H., and Cove, D.J. (1979). Analysis of gametophytic development in the moss, *Physcomitrella patens*, using auxin and cytokinin resistant mutants. Planta *144*, 427–435.
8. Prigge, M.J., Lavy, M., Ashton, N.W., and Estelle, M. (2010). *Physcomitrella patens* auxin-resistant mutants affect conserved elements of an auxin-signaling pathway. Curr. Biol. *20*, 1907–1912.
9. Grimsley, N.H., Ashton, N.W., and Cove, D.J. (1977). The production of somatic hybrids by protoplast fusion in the moss, *Physcomitrella patens*. Mol. Gen. Genet. *154*, 97–100.
10. Spinner, L., Pastuglia, M., Belcram, K., Pegoraro, M., Goussot, M., Bouchez, D., and Schaefer, D.G. (2010). The function of TONNEAU1 in moss reveals ancient mechanisms of division plane specification and cell elongation in land plants. Development *137*, 2733–2742.
11. Perroud, P.F., Demko, V., Johansen, W., Wilson, R.C., Olsen, O.A., and Quatrano, R.S. (2014). Defective Kernel 1 (DEK1) is required for three-dimensional growth in *Physcomitrella patens*. New Phytol. *203*, 794–804.
12. Demko, V., Perroud, P.F., Johansen, W., Delwiche, C.F., Cooper, E.D., Remme, P., Ako, A.E., Kugler, K.G., Mayer, K.F., Quatrano, R., and Olsen, O.A. (2014). Genetic analysis of DEFECTIVE KERNEL1 loop function in three-dimensional body patterning in *Physcomitrella patens*. Plant Physiol. *166*, 903–919.
13. Kim, D.-Y., Scalf, M., Smith, L.M., and Vierstra, R.D. (2013). Advanced proteomic analyses yield a deep catalog of ubiquitylation targets in *Arabidopsis*. Plant Cell *25*, 1523–1540.
14. Johansen, W., Ako, A.E., Demko, V., Perroud, P.-F., Rensing, S.A., Mekhlif, A.K., and Olsen, O.-A. (2016). The DEK1 calpain linker functions in three-dimensional body patterning in *Physcomitrella patens*. Plant Physiol. *172*, 1089–1104.

15. Girod, P.A., Fu, H., Zryd, J.P., and Vierstra, R.D. (1999). Multiubiquitin chain binding subunit MCB1 (RPN10) of the 26S proteasome is essential for developmental progression in *Physcomitrella patens*. Plant Cell *11*, 1457–1472.
16. Schuessele, C., Hoernstein, S.N.W., Mueller, S.J., Rodriguez-Franco, M., Lorenz, T., Lang, D., Igloi, G.L., and Reski, R. (2016). Spatio-temporal patterning of arginyl-tRNA protein transferase (ATE) contributes to gametophytic development in a moss. New Phytol. *209*, 1014–1027.
17. McKenna, A., Hanna, M., Banks, E., Sivachenko, A., Cibulskis, K., Kernytsky, A., Garimella, K., Altshuler, D., Gabriel, S., Daly, M., and DePristo, M.A. (2010). The Genome Analysis Toolkit: a MapReduce framework for analyzing next-generation DNA sequencing data. Genome Res. *20*, 1297–1303.
18. Li, H. (2013). Aligning sequence reads, clone sequences and assembly contigs with BWA-MEM. arXiv, arXiv:1303.3997v2, http://arxiv.org/abs/1303.3997.
19. Emms, D.M., and Kelly, S. (2015). OrthoFinder: solving fundamental biases in whole genome comparisons dramatically improves orthogroup inference accuracy. Genome Biol. *16*, 157.
20. Hiwatashi, Y., Obara, M., Sato, Y., Fujita, T., Murata, T., and Hasebe, M. (2008). Kinesins are indispensable for interdigitation of phragmoplast microtubules in the moss *Physcomitrella patens*. Plant Cell *20*, 3094–3106.
21. Engel, P.P. (1968). The induction of biochemical and morphological mutants in the moss *Physcomitrella patens*. Am. J. Bot. *55*, 438–446.
22. Nishiyama, T., Hiwatashi, Y., Sakakibara, I., Kato, M., and Hasebe, M. (2000). Tagged mutagenesis and gene-trap in the moss, *Physcomitrella patens* by shuttle mutagenesis. DNA Res. *7*, 9–17.
23. Frangedakis, E., Saint-Marcoux, D., Moody, L.A., Rabbinowitsch, E., and Langdale, J.A. (2017). Nonreciprocal complementation of KNOX gene function in land plants. New Phytol. *216*, 591–604.
24. Schaefer, D., Zryd, J.P., Knight, C.D., and Cove, D.J. (1991). Stable transformation of the moss *Physcomitrella patens*. Mol. Gen. Genet. *226*, 418–424.
25. Cove, D.J., Perroud, P.F., Charron, A.J., McDaniel, S.F., Khandelwal, A., and Quatrano, R.S. (2009). Somatic hybridization in the moss *Physcomitrella patens* using PEG-induced protoplast fusion. Cold Spring Harb. Protoc. *2*, pdb.prot5141.
26. Yasumura, Y., Moylan, E.C., and Langdale, J.A. (2005). A conserved transcription factor mediates nuclear control of organelle biogenesis in anciently diverged land plants. Plant Cell *17*, 1894–1907.
27. Bolger, A.M., Lohse, M., and Usadel, B. (2014). Trimmomatic: a flexible trimmer for Illumina sequence data. Bioinformatics *30*, 2114–2120.
28. Collingridge, P.W., and Kelly, S. (2012). MergeAlign: improving multiple sequence alignment performance by dynamic reconstruction of consensus multiple sequence alignments. BMC Bioinformatics *13*, 117.
29. Nguyen, L.-T., Schmidt, H.A., von Haeseler, A., and Minh, B.Q. (2015). IQ-TREE: a fast and effective stochastic algorithm for estimating maximum-likelihood phylogenies. Mol. Biol. Evol. *32*, 268–274.
30. Letunic, I., and Bork, P. (2016). Interactive tree of life (iTOL) v3: an online tool for the display and annotation of phylogenetic and other trees. Nucleic Acids Res. *44* (W1), W242–W245.
31. Livak, K.J., and Schmittgen, T.D. (2001). Analysis of relative gene expression data using real-time quantitative PCR and the 2(-Δ Δ C(T)) method. Methods *25*, 402–408.

STAR★METHODS

KEY RESOURCES TABLE

REAGENT or RESOURCE	SOURCE	IDENTIFIER
Antibodies		
Anti Digoxygenin AP Fab Fragments	Sigma-Aldrich	Cat#11093274910
Bacterial and Virus Strains		
E. coli strain DH5α	Widely distributed	N/A
Chemicals, Peptides, and Recombinant Proteins		
6-Benzylaminopurine (BAP)	Sigma-Aldrich	Cat#B3408
1-Naphthaleneacetic acid (NAA)	Sigma-Aldrich	Cat#N0640
Propidium Iodide (PI)	Sigma-Aldrich	Cat#P4864
G418 disulphate	Melford	Cat#G0175
Hygromycin B	Melford	Cat#H7502
Zeocin	ThermoFisher	Cat#R25001
Driselase Basidiomycetes sp.	Sigma-Aldrich	Cat#D8037
Poly(ethylene glycol) BioUltra 6,000 (PEG)	Sigma-Aldrich	Cat#81255
Critical Commercial Assays		
GoTaq G2 DNA Polymerase	Promega	Cat#M7841
Phusion High-Fidelity DNA Polymerase	ThermoFisher	Cat#F530S
TURBO DNA-free Kit	Ambion	Cat#AM1907
Superscript III Reverse Transcriptase	Invitrogen	Cat#18080093
SYBR Green PCR Master Mix	Applied Biosystems	Cat#4309155
Experimental Models: Organisms/Strains		
Physcomitrella patens subsp. *patens*	Widely distributed	N/A
Physcomitrella patens Vx::mCherry	Pierre-François Perroud	[5]
Physcomitrella patens Gd::GFP	Pierre-François Perroud	[5]
Ppnog1-R	This paper	N/A
Ppnog1/Gd-2	This paper	N/A
Ppnog1/Gd-3	This paper	N/A
Ppnog1/Gd-8	This paper	N/A
Ppnog1-R+pAct::PpNOG1:GFP-4	This paper	N/A
Ppnog1-R+pAct::PpNOG1:GFP-7	This paper	N/A
Ppnog1-R+pAct::PpNOG1:GFP-73	This paper	N/A
Ppnog1-R+pAct::PpNOG1:GFP-77	This paper	N/A
Ppnog1-R+pAct::PpNOG1:GFP-80	This paper	N/A
Ppnog1-D1	This paper	N/A
Ppnog1-D2	This paper	N/A
Oligonucleotides		
See Data S2	N/A	N/A
Recombinant DNA		
pZAG1	Yuji Hiwatashi (gift)	N/A
pAct:*PpNOG1*-mGFP	This paper	N/A
*pNOG1:PpNOG1**-mGFPmutNPTII-3′	This paper	N/A
pGFPmutNPTII plasmid	Yuji Hiwatashi	[17]
Software and Algorithms		
BWA-MEM	Sourceforge	[18]
OrthoFinder	GitHub	[19]
GATK	Broad Institute	[20]

CONTACT FOR REAGENT AND RESOURCE SHARING

Further information and requests for resources and reagents should be directed to and will be fulfilled by the Lead Contact, Jane Langdale (jane.langdale@plants.ox.ac.uk). Please note that the transfer of transgenic *P. patens* lines will be governed by an MTA and will be dependent on appropriate import permits being acquired by the receiver.

EXPERIMENTAL MODEL AND SUBJECT DETAILS

Plants

Vx::mCherry and Gd::GFP marker lines were obtained from Pierre-François Perroud [5]. The Gransden strain *Physcomitrella patens* subsp. *patens* [21], Vx::mCherry and Gd::GFP marker lines, and somatic hybrids were grown and maintained under sterile conditions on BCD or BCDAT medium. BCD medium contained 250mg/L $MgSO_4.7H_2O$, 250mg/L KH_2PO_4 (pH6.5), 1010mg/L KNO_3, 12.5mg/L $FeSO_4.7H_2O$, 0.001% Trace Element Solution (TES – 0.614mg/L H_3BO_3, 0.055mg/L $AlK(SO_4)_2.12H_2O$, 0.055mg/L $CuSO_4.5H_2O$, 0.028mg/L KBr, 0.028mg/L LiCl, 0.389mg/L $MnCl_2.4H_2O$, 0.055mg/L $CoCl_2.6H_2O$, 0.055mg/L $ZnSO_4.7H_2O$, 0.028mg/L KI and 0.028mg/L $SnCl_2.2H_2O$) and 8g/L agar, supplemented with 1 mM $CaCl_2$. BCDAT medium was additionally supplemented with 1 mM ammonium tartrate [22]. Plants were grown at 24°C with a 16 h: 8 h, light (300μmol m^{-2} s^{-1}): dark cycle. Sporophytes were induced as described previously [23] and harvested after approximately 3-6 months. Sporangia were sterilized in 20% Parozone bleach for 15 min at room temperature and then washed three times in sterile water. Spores were released using a sterile pipette tip and plated onto cellophane-overlaid BCDAT medium.

METHOD DETAILS

UV mutagenesis and screening

1% Driselase was prepared in 8% mannitol, incubated at room temperature for 15 min with gentle agitation, centrifuged for 3 min at 3,300 xg and then filter-sterilized (pellet discarded). 7-day old Vx::mCherry protonemata were harvested from cellophane-overlaid BCDAT medium and added to 1% Driselase and incubated, with gentle agitation, for 40 min. The cell suspension was then filtered through a sterile 50 μM filter into a round bottomed tube. The solution was centrifuged for 3 min at 120 xg at room temperature with no braking. Cells were then washed twice using 6 mL 8% mannitol and then resuspended in 6 mL 8% mannitol and cell density determined using a haemocytometer [24]. Protoplasts were plated at a density of $5x10^4$ cells mL^{-1}, onto cellophane overlaid BCDAT supplemented with 10 mM $CaCl_2$, 6% mannitol and 0.5% glucose (BCDATG medium). Protoplasts were then exposed to a 75000 μJ dose of UV radiation using a Stratalinker UV Crosslinker to achieve an 85%–90% kill rate (this will vary according to the equipment utilized and must be calibrated prior to screening). UV-treated protoplasts were incubated at 24°C in the dark for 48 h prior to growth under standard conditions for one week. Cellophane discs were then transferred to medium containing BCDAT medium and grown under standard conditions for a further week. Regenerating protoplasts were transferred to individual wells of 24-well plates containing BCD medium to induce gametophores. Regenerating mutagenized lines that did not produce gametophores after a period of two months were selected for further validation.

To determine whether 3D-defective mutants were cytokinin-responsive, protonemata were cultured on cellophane-overlaid BCDAT medium for four days under standard growth conditions. Cellophane discs were then transferred to BCDAT medium containing 5 μM benzylaminopurine (BAP) and cultivated for 3 d.

Microscopy

Fluorescence microscopy was carried out with a Zeiss LSM510 META confocal microscope. A 40x water immersive lens (C-Apochromat 40x/1.20 W) was used for all imaging. Propidium iodide (PI) was excited at 488 nm with 15% laser power and detected with a 565-615 bandpass filter. Tissues were carefully removed from cellophane-overlaid BCD plates, submerged in 10 μgmL^{-1} PI for 1 min and then mounted on slides in water. Other images were captured using either a Leica DMRB microscope or a Leica M165C microscope, equipped with a QImaging Micropublishing 5.0 RTV camera.

Somatic hybridization and segregation analysis

Protoplasts were isolated from *nog1-R* and Gd::GFP as described above. The cell suspensions derived from each strain were adjusted to a density of $1x10^6$ cells mL^{-1} in 8% mannitol. 1 mL of each strain were combined, mixed gently and then centrifuged for 3 min at 120xg at room temperature with no braking. Protoplasts were resuspended in 250 μL PW solution (10 mM $CaCl_2$ and 8.5% mannitol). Somatic hybridization was initiated by adding 750 μL PEG/F (5 mM $CaCl_2$ and 50% PEG 6000 (w/v)) and mixed gently. After 40 min, 1.5 mL of PW solution was added. After 50 min, 10 mL of PW solution was added. After 60 min, 10 mL of PW solution was added. Following each addition of PW solution, protoplasts were mixed gently. After 70 min, protoplasts were centrifuged at 120 xg for 3 min at room temperature with no braking. The pellet was resuspended in 4 mL 8% mannitol and then 1 mL each plated onto cellophane-overlaid BCDATG medium [25]. Plates were kept in the dark for 48 h and then transferred to standard growth conditions. After one week, cellophane discs were removed and then transferred to BCDAT medium supplemented with both 50 μgmL^{-1} G418 and 20 μgmL^{-1} hygromycin B, to select for stable *Ppnog1-R*/Gd::GFP diploid hybrids.

Sporophytes were induced as above and the resulting spore progeny allowed to grow on BCDAT for two weeks. Segregation analysis was carried out by transferring sporelings to individual wells of a 24-well tissue culture plate containing BCD medium for two months, and then scoring phenotypes.

Isolation of genomic DNA and sequencing

Total genomic DNA was isolated from one week old protonemata maintained on cellophane-overlaid BCDAT medium using the CTAB method [26]. Essentially, tissue was first ground in liquid nitrogen, and suspended in 500 μL CTAB extraction solution (1.5% CTAB, 1.05 M NaCl, 75 mM Tris-HCl, 15 mM EDTA pH8.0). After incubation at 65°C for 10 min, an equal volume of chloroform:isoamylalcohol (24:1) was added, samples were mixed by vortexing and then centrifuged at 13,000 rpm for 10 min. The upper aqueous layer of each sample was transferred to a fresh tube. DNA was precipitated by adding 0.7 volumes of isopropanol, and centrifuged at 13,000 rpm for 10 min. Pellets were washed with 500 μL 70% ethanol, and air-dried. DNA samples were dissolved in 50 μL H_2O and stored at −20°C. Genomic DNA extracted from individual protonemal cultures was pooled in equimolar amounts to construct separate mutant (120 individuals; 1.7 μg total) and wild-type (120 individuals; 3.5 μg total) pools. Genomic DNA was extracted from both Vx::mCherry (2.6 μg total) and Gd::GFP (3.5 μg total) parental lines to identify SNPs from both genetic backgrounds for use in bulk segregant analysis. DNA samples were sequenced using an Illumina HiSeq4000 platform (150 bp PE read lengths) at the Wellcome Trust Centre for Human Genetics, University of Oxford.

Read processing and variant calling

Raw reads were subject to quality filtering using Trimmomatic [27] to remove low quality bases, read-pairs and contaminating adaptor sequences prior to read mapping and variant calling. Sequences were searched for all common Illumina adaptors and the settings used for read processing by Trimmomatic were LEADING:20 TRAILING:20 SLIDINGWINDOW:5:20 MINLEN:50. Each of the trimmed quality filtered paired-end read libraries were mapped using BWA-MEM [18] to the *P. patens* genome (Version Ppatens_318_v3) obtained from Phytozome V11. Duplicate mapped reads were removed, mapped reads were realigned around indels, and variants called according to best practice guidelines from GATK using GATK v3.6 [20].

Candidate gene discovery through bulk segregant analysis

Comparison of the sequence data from the Gd::GFP and Vx::mCherry parental strains identified a set of 2,255,671 single nucleotide variants that distinguished the two strains. To conduct the bulk segregant analysis, the reads from the wild-type and mutant pools were mapped to the reference genome and the allele frequency of the strain-distinguishing variants in both pools was evaluated. Strain-distinguishing variants were used for analysis if: 1) the coverage depth in both the wild-type and mutant pools was > 0.5 and < 2 times the mean coverage depth and; 2) the variant quality was > 500. These allele frequencies were used to identify the chromosomal region linked to the mutant allele. To confirm the presence of the premature termination codon in the *PpNOG1* gene in the *Ppnog1-R* mutant, the full-length transcript was amplified using the primers 32970008(exon)_GSPFSalI and 32970008(exon)_GSPR*Hind*III (Data S2), and sequenced.

Physcomitrella patens transformation

2g polyethylene glycol 6000 (previously autoclaved in flat-bottomed autoclavable vial) was melted using a microwave. 5 mL mannitol/$Ca(NO_3)_2$ solution (0.8% mannitol, 0.1 M $Ca(NO_3)_2$, 10 mM Tris pH8.0) was then added to the molten PEG and incubated for 2-3 hours at room temperature. Protoplasts were isolated from both *nog1-R* and Vx::mCherry as described above. However, following cell density determination, protoplasts were resuspended in MMM (0.5 M mannitol, 0.15 M $MgCl_2$, 0.1% MES pH5.6) to achieve a final cell density of $1.5x10^6$ cells mL^{-1}. 10 μg of the linearized construct was added to a round bottomed tube. 300 μL of protoplast suspension and 300 μL PEG solution were then added to the tube in drops and mixed gently. The samples were heat-shocked for 5 min at 45°C and incubated for an additional 5 min at room temperature. 300 μL 8% mannitol was added to each tube, 5 times at 3 min intervals and tilted gently to mix after each addition. 1 mL 8% mannitol was then added to each tube, 5 times at 3 min intervals and tilted gently to mix after each addition. Cells were centrifuged for 4 min at 120 xg, supernatants discarded and pellets resuspended in 3 mL 8% mannitol. 1 mL was plated onto each of three cellophane-overlaid BCDATG plates, which were subsequently sealed and kept in the dark for 48 h. Plates were transferred to standard growth conditions for a further 5 days [24]. Cellophane discs were then removed and transferred to BCDAT medium supplemented with either 50 μgmL^{-1} G418 or 100 μgmL^{-1} Zeocin.

After two weeks, plants were transferred back to non-selective BCDAT medium for another week and then stable transformants were selected by plating on selective medium again for a further week. 10 μg plasmid DNA was transformed into protoplasts. Stable transgenic lines were analyzed by PCR (using primers in Data S2) and DNA gel blot analysis (see below).

Generation of nog1-R complementation lines

The full-length *PpNOG1* coding sequence (excluding the stop codon) was amplified from *P. patens* Gransden wild-type protonemata cDNA using the primers 32970008(exon)_GSPFSalI and 32970008(exon)_GSPR*Hind*III (Data S2) and ligated into SalI/*Hind*III cut pZAG1 plasmid (a gift from Yuji Hiwatashi). The resultant construct, pAct:*PpNOG1*-mGFP (Data S1A), was linearized using KpnI and transformed into protoplasts isolated from *Ppnog1-R* mutants. Stable transformants were selected using 100 μgmL^{-1} Zeocin.

Generation of a nog1 disruptant mutant

A genomic DNA fragment from - 827bp upstream of the start codon up to but excluding the start codon of the *PpNOG1* sequence was PCR-amplified using 32970008_KI.5FKpnI_revised and 32970008_KI.5R*Xho*I primers (Data S2) and ligated into KpnI/*Xho*I cut pGFPmutNPTII plasmid [17] to create *pNOG1*-mGFPmutNPTII. A *PpNOG1* genomic DNA fragment including the stop codon up to 1420bp downstream of the *PpNOG1* stop codon was PCR-amplified using 32970008_KI.3F*Not*I and 32970008_KI.3R*Sac*II primers (Data S2) and ligated into *Not*I/*Sac*II cut *pNOG1*-mGFPmutNPTII to create *pNOG1*-mGFPmutNPTII-3′. The *PpNOG1* coding sequence was PCR-amplified from cDNA prepared from the *nog1-R* mutant using 32970008(exon)_GSPFSalI and 32970008(exon)_GSPR*Hind*III primers (Data S2) and then ligated into SalI/*Hind*III cut *pNOG1*-mGFPmutNPTII-3′ to create *pNOG1*:*PpNOG1**-mGFPmutNPTII-3′ (Data S1B). *pNOG1*:*PpNOG1**-mGFPmutNPTII-3′ was linearized using KpnI before being transformed into protoplasts isolated from Vx::mCherry. Stable transformants were selected using 50 μgmL^{-1} G418.

DNA gel blot analysis

15 μg genomic DNA was digested with *Hind*III for 3 h at 37°C, according to the manufacturer's instructions (NEB), and then electrophoresed on a 1% agarose gel. The gel was treated with depurination solution (0.25 M HCl) for 20 min, denaturing solution (1.5 M NaCl, 0.5 M NaOH) for 30 min, neutralization solution (1M Tris.Cl pH8.0, 1.5 M NaCl) for 30 min and then soaked in 2xSSC before being incorporated into the blotting apparatus and the DNA transferred to N$^+$ Hybond membrane (Amersham). The nylon membrane was rinsed in 2xSSC and then UV-crosslinked using a Stratalinker.

The pNOG1 fragment for hybridization was PCR amplified from *pNOG1*:*PpNOG1**-mGFPmutNPTII-3′ using GoTaq Flexi DNA Polymerase (Promega), but with the addition of 0.25 mM Digoxygenin-11-dUTP (Roche) (Data S2).

Membranes were incubated in DIG Easy Hyb^{TM} (Roche) for 2 h at 58°C. 20 μg sheared salmon sperm and 30 ng purified probe were added to 50 μL water (per 15 mL of DIG Easy Hyb^{TM} solution), boiled at 100°C for 5 min and then allowed to cool before adding to the DIG Easy Hyb^{TM} solution. Blots were hybridized overnight at 58°C and then washed twice for 5 min in 2xSSC, 0.1% SDS at room temperature, and twice for 30 min in 0.5xSSC, 0.1% SDS at 58°C. Membranes were incubated in 1xMaleic Acid Buffer (0.1 M Maleic Acid, 0.15 M NaCl) for 3 min and subsequently in blocking buffer (5% skimmed milk powder in 1xMaleic Acid Buffer) for 2 h at room temperature. Blocking buffer was then replaced with 50 mL blocking buffer containing 3.75 U Anti Digoxygenin AP Fab Fragments (Roche) and blots incubated for 30 min. Blots were washed with 1xWashing Buffer (0.1xMaleic Acid Buffer + 0.3% Tween 20) three times for 15 min and once in 1xDetection Buffer (0.1 M Tris-HCl, 0.1 M NaCl, pH9.5) for 3 min. Membranes were incubated in the presence of 5:1000 dilution of CDP-Star Detection reagent (manufacturer) in a sheet protector for 5 min and then exposed to film in an autoradiography cassette.

Quantitative RT-PCR

Total RNA was isolated using the RNeasy Mini kit (QIAGEN) and DNase treated (Turbo DNase, Ambion). cDNA was synthesized using Superscript III Reverse Transcriptase (Invitrogen) and 500 ng DNase-treated RNA. For quantitative RT-PCR, primer pairs were designed to amplify ~150bp fragment of each of the genes of interest. Amplification was detected using SYBR Green (Life Technologies) on a StepOne^{TM} Real-Time PCR System (Applied Biosystems). Cycling conditions were 95°C for 5 min, and 40 cycles of 95°C for 15 s and 60°C for 1 min. To test for genomic DNA contamination, no RT controls were included. To test responsiveness of *PpNOG1* to cytokinin and auxin, 7 d old wild-type Gd protonemata were transferred to BCD medium supplemented with 1 μm BAP, 1 μm NAA or 1 μm BAP/1 μm NAA (plus controls), and grown for an additional 3 d. To quantify *PpAPB* transcripts, RNA was isolated from 14 d old protonemata (Vx::mCherry and *Ppnog1-D1*) grown on cellophane-overlaid BCD medium.

Phylogenetic analysis

The complete set of predicted proteomes for all species in Phytozome version 10 were subject to orthogroup inference using OrthoFinder [19]. The orthogroup containing the NOG1 gene was identified and the constituent sequences subject to multiple sequence alignment using MergeAlign [28]. It was apparent from manual inspection of the multiple sequence alignment that several gene models were likely incorrect (*Spirodella polyriza, Vitis vinifera, Eucalyptus gradis, Setaria italica, Gossypium raimondii*) or potentially absent (*Selaginella moellendorfii*). Gene models were corrected or predicted manually and added to the multiple sequence alignment. Further sequences were added to the alignment after a manual BLAST search of Phytozome version 12 (Data S3A). After empirical testing of the multiple sequence alignment for maximum likelihood phylogenetic tree inference using IQTREE [29], a subset of sequences was selected, retaining representatives of genera in all phyla and evidence of gene duplications where present, while eliminating sequences that are likely mis-annotated (Data S3B). The best-fitting model parameters (JTT+G4) were estimated from the revised alignment (Data S4) and a consensus phylogenetic tree was estimated from 1000 bootstrap replicates. The data were imported into ITOL [30] to generate the pictorial representation.

QUANTIFICATION AND STATISTICAL ANALYSES

Experimental design, sampling and statistical methods

The number of gametophores per culture was quantified by counting the number of gametophores derived from a homogenized culture of protonemata. Cultures were normalized to a uniform OD so that direct comparisons could be made between wild-type

and transgenic lines. For all experiments, at least ten individuals representing more than two independent transgenic lines were evaluated. For all counts, the Standard Error of the Mean was determined and a t test was performed using Excel.

qRT-PCR experiments were carried out using three technical replicates for each of three independent biological samples, alongside water controls. Ct values were calculated from raw amplification data using the Real-time PCR Miner software (http://www.ewindup.info/miner/). The mean Ct value between the three technical replicates was then calculated. Fold changes in gene expression were calculated relative to controls using the $2^{-\Delta CT}$ method [31]. Two or three genes were used as constitutive controls: EF1alpha (Pp1s84_186V6), DNAJ (Pp1s123_116V6), ACT7 (Pp1s198_154V6). An ANOVA test was performed to compare whether relative change was statistically significant.

A phenotypic segregation analysis was performed on the progeny derived from three independent *Ppnog1-R*/Gd hybrid sporophytes. At least 570 individuals were scored per line and a Chi-square test was performed to check for conformity to a 1:4:1 segregation ratio expected from meiosis from a tetraploid.

DATA AND SOFTWARE AVAILABILITY

The accession number for the raw genome sequence reads reported in this paper is EBI ArrayExpress: E-MTAB-5096. The accession numbers for the sequences of full length *PpNOG1* cDNA plus the three splice variants in *nog1-R* mutants reported in this paper are NCBI: MG280833, MG280834, MG280835, and MG280836, respectively.

Current Biology, Volume *28*

Supplemental Information

Genetic Regulation of the 2D to 3D Growth Transition in the Moss *Physcomitrella patens*

Laura A. Moody, Steven Kelly, Ester Rabbinowitsch, and Jane A. Langdale

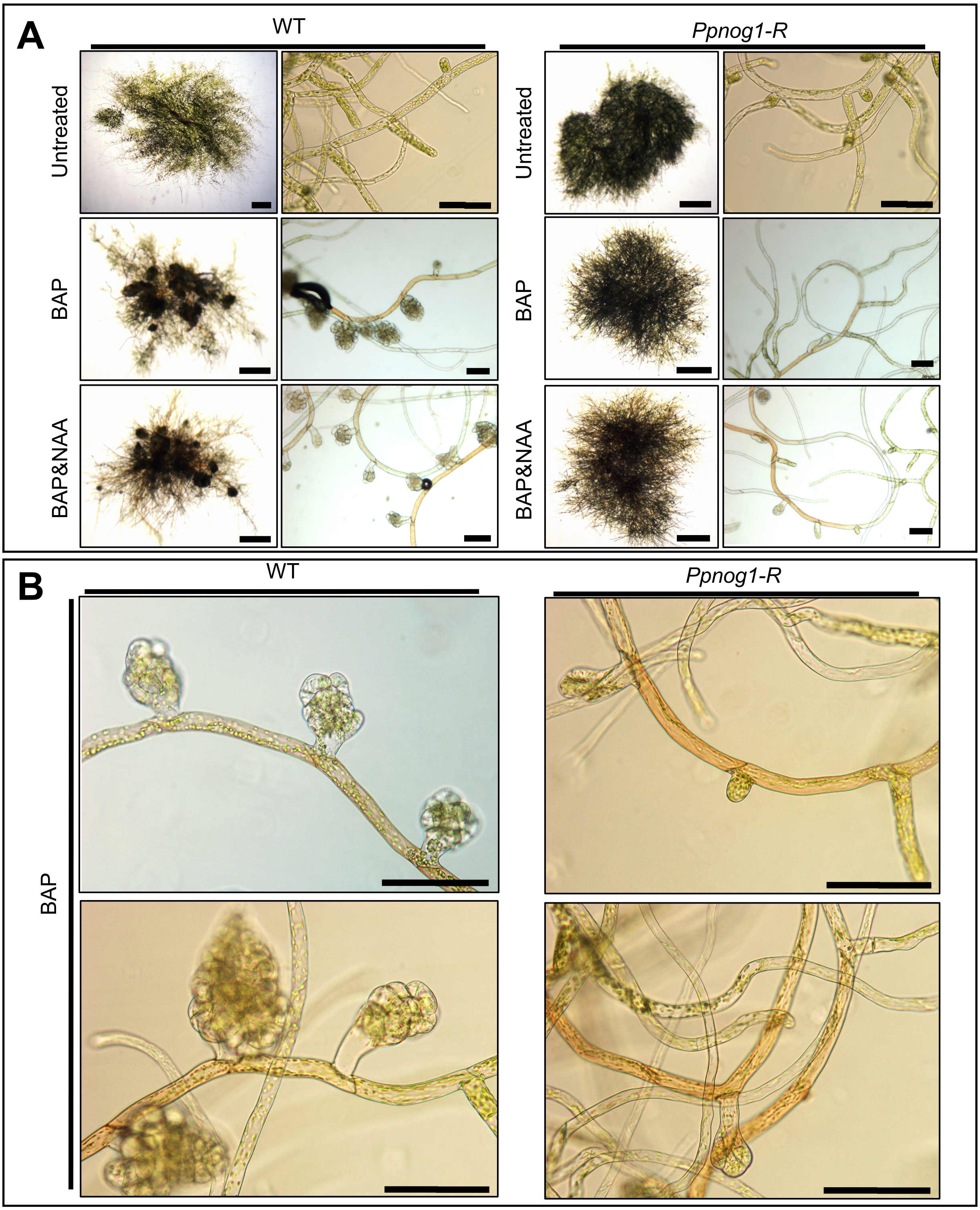

Figure S1. Gametophores are not induced by cytokinin in *Ppnog1-R* mutants. Related to Figure 1. A) 7d old wild-type (WT) and *Ppnog1-R* protonemal filaments cultured in the presence or absence of 1 μm BAP and/or 1 μm NAA. Scale bars = 1 mm. **B)** High magnification images of BAP-treated WT and *Ppnog1-R* protonemal filaments. Scale bars = 50 μm.

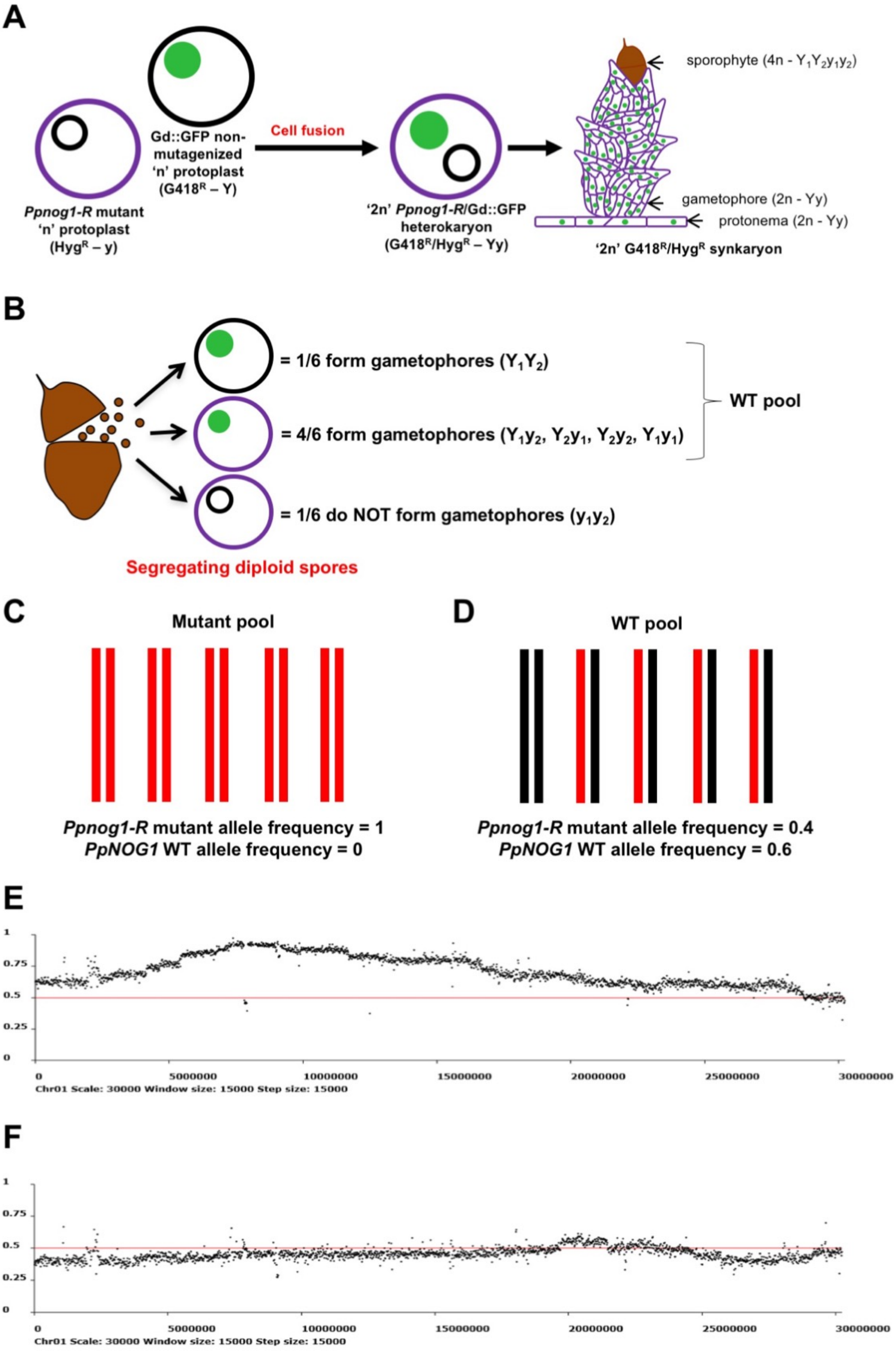

Figure S2. Bulk segregant analysis via somatic hybridization and identification of the *PpNOG1* locus. Related to Figure 2. A) Somatic hybridization between reproductively sterile *Ppnog1-R* (haploid, n) and Gd::GFP (n) protoplasts yields viable *Ppnog1-R*/Gd::GFP diploid hybrid protoplasts which regenerate to form diploid protonema and gametophores, that produce tetraploid sporophytes after fertilization. **B)** *Ppnog1-R*/Gd::GFP tetraploid sporophyte undergoes meiosis to produce phenotypically segregating diploid spores. **C, D)** Segregating progeny sequenced in separate mutant (C) and wild-type pools (D). Expected *Ppnog1-R* and *NOG1* allele frequencies in each pool are indicated. **E, F)** Allele frequency plots for *Ppnog1-R* (E) and wild-type (F) segregants along chromosome 1 of *Physcomitrella patens*.

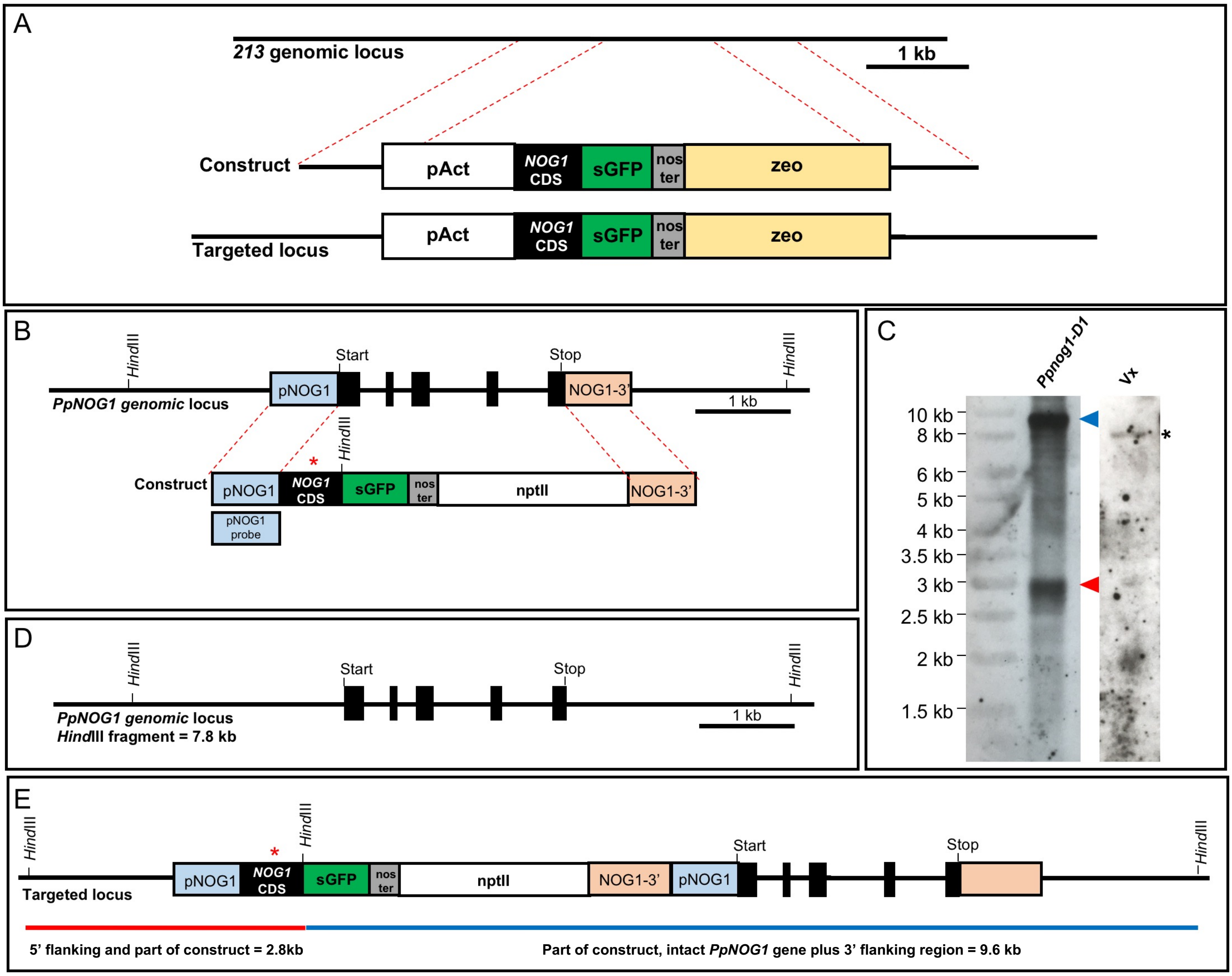

Figure S3. Generation of *PpNOG1* complementation and disruptant lines. Related to Figure 3. A) Schematic of the construct used to complement the *Ppnog1-R* mutant phenotype. **B)** Schematic of the construct used to disrupt the *PpNOG1* locus in the Vx parental line. The *pNOG1* probe used in (C) is indicated. **C)** DNA gel blot analysis of the disruptant line. Genomic DNA from Vx parental and *Ppnog1-D1* disruptant lines was digested with HindIII and hybridized with the *pNOG1* probe (B). An asterisk (*) denotes the presence of an intact *PpNOG1* locus in Vx (detailed in D) and coloured arrows refer to two separate fragments from the disrupted *PpNOG1* locus (detailed in E). **D)** Schematic of the *PpNOG1* locus in parental Vx (* in C). **E)** Schematic of *PpNOG1* locus in the disruptant line *Ppnog1-D1* (coloured arrows in C). Expected fragment sizes are indicated, which are supported by sequencing data (data not shown).

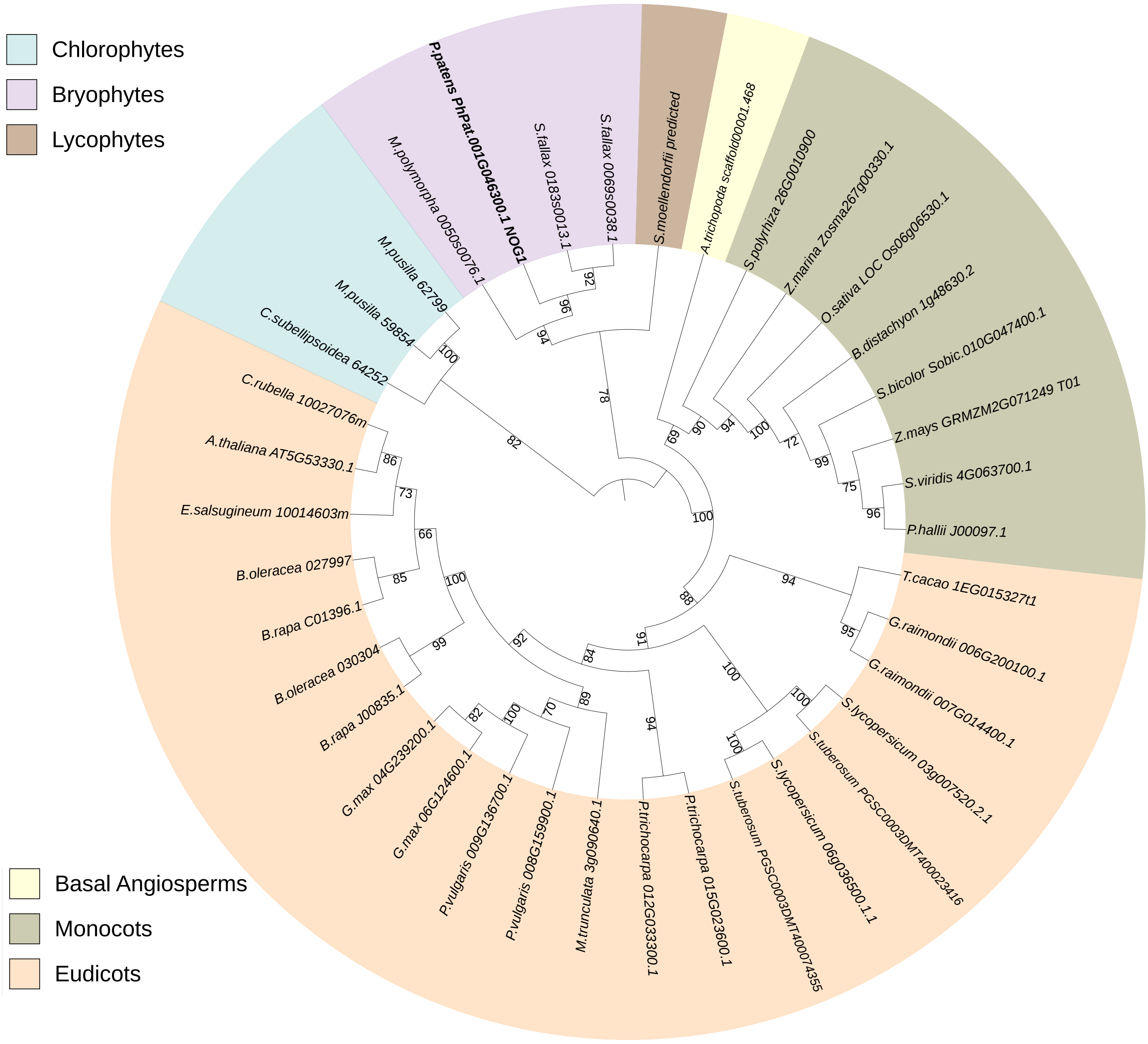

Figure S4. Maximum-likelihood phylogenetic analysis of NOG1 homologs. Related to Figure 4. Bootstrap values are indicated next to the corresponding branch. Colour coded boxes correspond to the species phyla. The tree was rooted using sequences from the chlorophyte clade that share homology in the ubiquitin-associated domain but are otherwise dissimilar (see Data S4 for sequence alignment).
